# The Temozolomide–Doxorubicin paradox in Glioblastoma in vitro–in silico preclinical drug-screening

**DOI:** 10.1038/s41598-024-53684-y

**Published:** 2024-02-14

**Authors:** Mariam-Eleni Oraiopoulou, Eleftheria Tzamali, Stylianos E. Psycharakis, Georgios Tzedakis, Takis Makatounakis, Katina Manolitsi, Elias Drakos, Antonis F. Vakis, Giannis Zacharakis, Joseph Papamatheakis, Vangelis Sakkalis

**Affiliations:** 1 Institute of Computer Science (ICS), Foundation for Research and Technology-Hellas (FORTH), Heraklion, Greece; 2 Present Address: Cancer Research UK – Cambridge Institute, University of Cambridge, Cambridge, UK; 3 Institute of Electronic Structure and Laser (IESL), Foundation for Research and Technology Hellas (FORTH), Heraklion, Greece; 4 School of Medicine, University of Crete, Heraklion, Greece; 5 Institute of Molecular Biology and Biotechnology (IMBB), Foundation for Research and Technology Hellas (FORTH), Heraklion, Greece; 6 University General Hospital of Heraklion (PAGNI), Heraklion, Greece; 7 Department of Biology, University of Crete, Heraklion, Greece

**Keywords:** Brain cancer, Preclinical drug-screening, Computational models, Temozolomide, Doxorubicin, Chemotherapy, Computational models, Cancer models

## Abstract

Adjuvant Temozolomide is considered the front-line Glioblastoma chemotherapeutic treatment; yet not all patients respond. Latest trends in clinical trials usually refer to Doxorubicin; yet it can lead to severe side-effects if administered in high doses. While Glioblastoma prognosis remains poor, little is known about the combination of the two chemotherapeutics. Patient-derived spheroids were generated and treated with a range of Temozolomide/Doxorubicin concentrations either as monotherapy or in combination. Optical microscopy was used to monitor the growth pattern and cell death. Based on the monotherapy experiments, we developed a probabilistic mathematical framework in order to describe the drug-induced effect at the single-cell level and simulate drug doses in combination assuming probabilistic independence. Doxorubicin was found to be effective in doses even four orders of magnitude less than Temozolomide in monotherapy. The combination therapy doses tested in vitro were able to lead to irreversible growth inhibition at doses where monotherapy resulted in relapse. In our simulations, we assumed both drugs are anti-mitotic; Temozolomide has a growth-arrest effect, while Doxorubicin is able to cumulatively cause necrosis. Interestingly, under no mechanistic synergy assumption, the in silico predictions underestimate the in vitro results. In silico models allow the exploration of a variety of potential underlying hypotheses. The simulated-biological discrepancy at certain doses indicates a supra-additive response when both drugs are combined. Our results suggest a Temozolomide–Doxorubicin dual chemotherapeutic scheme to both disable proliferation and increase cytotoxicity against Glioblastoma.

## Introduction

Glioblastoma multiforme or glioblastoma (GB) brain cancer treatment primarily entails Temozolomide (TMZ) chemotherapy and maximal safe surgical resection and/or radiotherapy. The high inter- and intra-tumoral heterogeneity and post-surgery relapse, often do not result in good prognosis for the patient. Half of the TMZ-treated GB patients do not positively respond to treatment^1^, yet to date, no other chemotherapeutic against primary or recurrent GB has been reported to be more effective. GB recent trends in preclinical^2–4^ and clinical^5–7^ studies usually imply Doxorubicin (DOX); one of the oldest (breast) cancer chemotherapeutics which has been also FDA-approved (Food and Drug Administration). Currently, there are active clinical trials worldwide recruiting brain cancer and especially glioma and GB eligible patients to test DOX-derivatives and DOX-excipients either adjuvantly or as a monotherapy (for example, study numbers NCT02372409 and NCT05630209 on clinicaltrials.gov).

TMZ is a lipophilic imidazotetrazine derivative of a DNA-alkylating agent (dacarbazine) which was firstly introduced in the 1970’s to target melanoma^8^. Since 2005, TMZ is used in GB therapy^9^ because of its ability to overpass the restrictive blood brain barrier (BBB) with a bioavailability of 98% when orally administrated^10^. In most of the cases, TMZ blocks cell division process by disrupting the DNA replication and subsequently causes various cell cycle phase arrest accompanied by accumulation of DNA lesions, possibly resulting in cell death. TMZ-induced cell death may also vary between senescence or autophagy preceding apoptosis, but not necrosis which comprises less than 5% of cell death^11^. TMZ-resistance has been correlated to O^6^ methyltransferase (MGMT) overexpression and/or malfunctioning DNA repair mechanisms, such as mismatch repair inhibition, that prohibit DNA damage accumulation^1,12^. Additionally, the MGMT promoter hypermethylation has been associated with enhanced TMZ responsiveness both in vitro and in clinic^8^.

DOX is an non-selective class I anthracycline antitumor antibiotic, extracted from *Streptomyces peucetius var. caesius*^13^. There has been reported acidic pH-related chemoresistance regarding DOX^14^. Only dividing tumor cells can be affected by DOX, hence it is considered a cell cycle-specific chemotherapeutic. DOX mechanism of action, although not fully understood, is related to: (i) DNA repair disruption mediated by the topoisomerase II resulting in a large number of DNA fragments, (ii) intercalation of genomic and mitochondrial DNA molecules inhibiting transcription, and (iii) increase of quinone type iron-mediated free radical production, which damages the cell structure up to cell death^13^, most usually necrosis 24 h post-treatment^15^ and even as a late effect^16^. As an anthracycline, DOX within the body is characterized by a rapid distribution phase and a slow elimination phase, but the drug’s distribution is slow within the tumor since it shows high affinity for the biomolecules. However, there is no need for excessive doses in order for DOX to access the inner tumor regions since it has been shown to be stored within the cell and re-released after cell death in a way that its administration/ distribution is spatiotemporally prolonged^17^. Low intravenous DOX administration prevents cardiotoxicity^18^. Especially regarding the brain interstitium, DOX is unable to overpass the BBB because of (i) high molecular weight, (ii) low lipophilicity and (iii) the cerebrospinal fluid-efflux due to the p-glycoprotein^18^. Thus, the main reason why DOX is not widely used in GB treatment is that in order to achieve adequately effective concentration within the brain via systematic administration, congestive heart failure is possible. Another unique characteristic of DOX is that, unlike other chemodrugs, it has autofluorescence (excitation: 480 nm, emission: 590 nm) often used to identify interaction with the cancer cells and estimate drug distribution^2^.

The idea of repurposing an old chemotherapeutic of wide applicability is not new and studies aim to either assess the anthracycline mechanism of action and toxicity to the GB cells^2,18,19^, a combined TMZ-DOX treatment option^2,6,20^ or the DOX/DOX-derivatives drug delivery potentials within the brain^3,18,21,22^ or the GB cells^2,23^. There is also recent literature concerning the in vivo combination of the two drugs, yet in cancer types other than GB^24–26^. Although conventional 2D in vitro end-point drug-screening assays provide valuable information regarding drug effectiveness and potency, they have thus far proven insufficient to predict the spatiotemporal response of tumors^27^. On the other hand, animal studies, though closer to human, are most usually time-consuming and cost-ineffective, while they fail to adequately identify drug molecular and mechanistic interactions. In an aim to diminish the differences between in vitro and in vivo preclinical drug studies, advanced approaches combining in vitro experiments with multiscale computational methods that account for the pharmacodynamics effect of each agent on each cancer cell and the spatiotemporal tumor complexity, do provide the means to more accurately evaluate the effectiveness of current or new treatments. Furthermore, through computational approaches, patient selection, drug selection and exposure schemes will be advanced, and overall clinical translatability will be enhanced in a time-efficient way. There are computational studies regarding the population dynamics and treatment response based on biological data dedicated to the simulation of drug combinations other than TMZ-DOX against GB progress^28^ or to other cancer types^29–31^. There is a long list of GB -specific computational models that have been in vivo validated with human or xenograft models based on other form of therapies, such as hypoxia-related death^32,33^, radiotherapy and/or combination therapies^34–36^, indicating the wide applicability of such a mechanistic approach. A variety of GB -specific TMZ^37–39^- or DOX-only^31,40,41^ simulations also exist. Thus far, little is known for the mechanistic dynamics of TMZ and DOX in monotherapy in 3D systems; let alone the mechanistic behavior of cells in the presence of both TMZ and DOX and whether their combination may provide any therapeutic benefit in GB therapy.

In this work, GB cells were treated with a wide range of TMZ and DOX concentrations, either as monotherapy or in combination. The biological monotherapy results were further used to parametrize a hybrid discrete–continuous (HDC) GB predictive computational algorithm. A probabilistic mathematical description was formulated to describe the mechanisms of action for each regime and predict a range of doses where the two drugs could act beneficially in combination, assuming probabilistic independence. We define as beneficial, doses aiming towards minimizing the drug doses, while keeping the high efficacy. Interestingly, under the non-synergistic assumption, the computational model’s predictions underestimated the respective in vitro results. Surprisingly, a supra-additive efficacy was observed in vitro indicating a potential TMZ-to-DOX or DOX-to-TMZ effect. Our overall results are in favor of a maximal efficacy-minimal dosing TMZ-DOX combined therapeutic scheme that disables proliferation and increases cytotoxicity against GB.

## Methods

### Cell cultures

All methods are in accordance with relevant institutional guidelines and regulations. Human sample was anonymously provided with the informed patient’s consent by the Neurosurgical Clinic of the University General Hospital of Heraklion (PAGNI), Crete, Greece under the research protocol number 442/12/02.05.2018. The study is conducted in accordance with the National Regulatory Framework and the provisions of Law 4521/2018 (Articles 21–27(E)—'Ethics Committees for Research’) and is approved by the Ethics Committee of PAGNI (‘Research Ethics Committee of PAGNI') and FORTH ('Research Ethics Committee of FORTH’).

**Figure 1:**
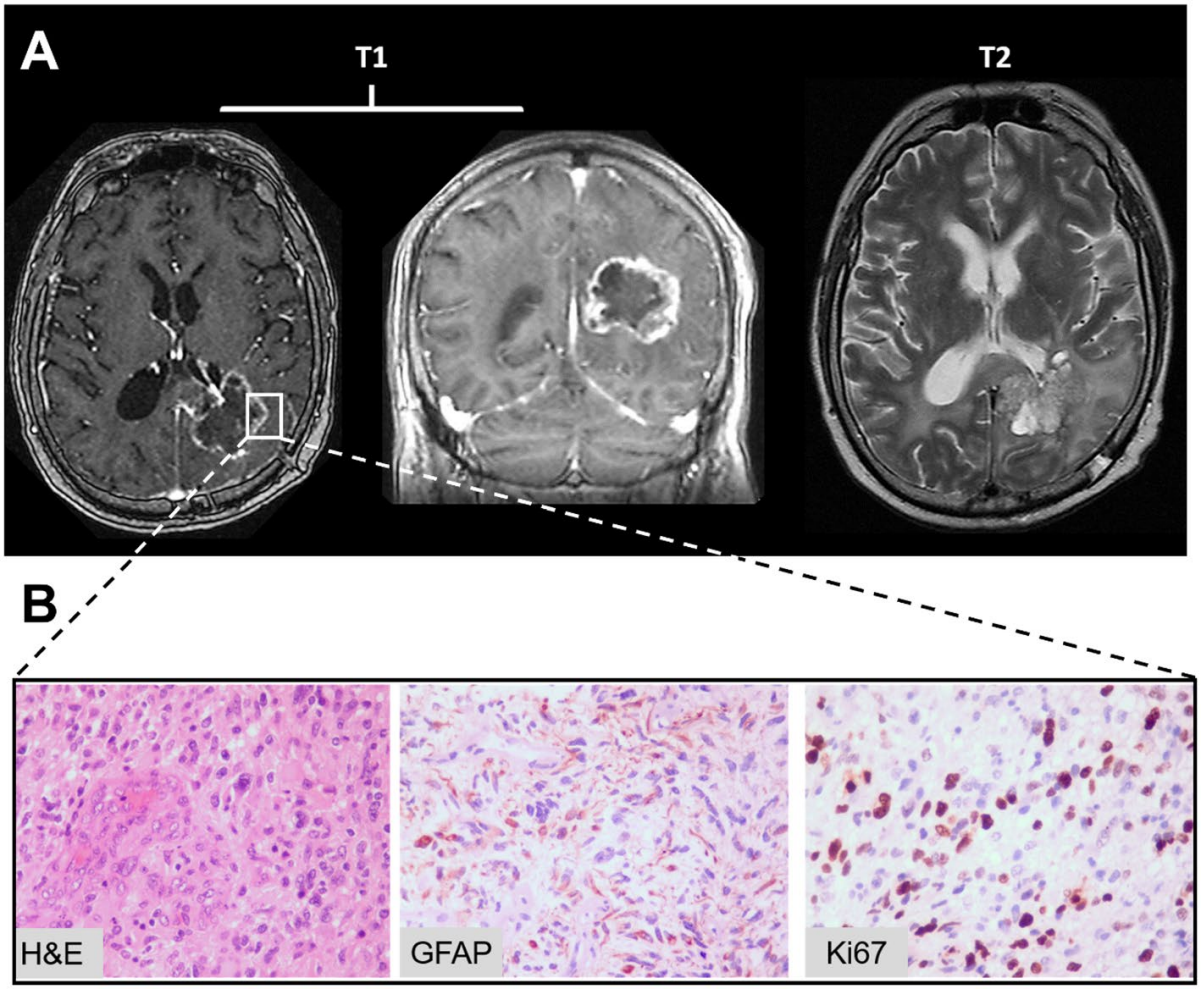
Clinicopathological results of the patient enrolled. (**A**) Lesion site of where the biopsy was taken from a 53-year-old male patient with GB in the temporal-occipital left hemisphere. Representative axial T1 multi-planar reformation (left) and coronal T1 (center), as well as T2 axial magnetic resonance images (right) are shown from the tumor central plane. Cells from the collected tissue sample were used to form the primary GBP08-P0 cell line. (**B**) GB biopsy. Hematoxylin and eosin (H&E) staining shows morphological characteristics. GFAP is a glial marker. Ki67 is a marker of proliferation. Original magnifications at 400x.

The own-established GBP08-P0 GB cell line was investigated and the U87MG (ATCC®, HTB-14™, USA) cells were used as a reference. Both cell lines were up to passage 20 and were grown in 5% CO_2_ using DMEM-F12 supplemented with 10% fetal bovine serum and 50 μg/ml gentamycin. The establishment of the primary cell line was performed as described in^42^. Brain cancer collected during the gross resection of a 53-year-old male patient with histopathologically-confirmed de novo temporo-occipital GB in the left hemisphere, as depicted in Fig. 1A. The sample collected originated from multiple parts of the tumor regions as assessed with intraoperative neuronavigation to avoid clonal selection during the establishment of the GBP08-P0 cell line.

### RNA extraction and qRT-PCR

Total RNA was extracted from 10^7^ cells using the Macherey–Nagel™ NucleoZOL (Fisher Scientific, UK). 2 mg RNA was used to generate cDNAs using the M-MuLV Reverse Transcriptase (Biolabs, UK) in the presence of RNase inhibitor (Biolabs, UK). The transcript abundance was measured by qRT-PCR using the dye SYBR Green I (Invitrogen, USA). Relative mRNA expression levels were calculated after normalization against GADPH. The primers used for qRT-PCR were 5′-CAGTCAGCCGCATCTTCTTT-3′ and 5′-ACCAGAGTTAAAAGCAGCCC-3′ for GADPH and 5′-GCTGAATGCCTATTTCCACC-3′ and 5′-ACAACCTTCAGCAGCTTCCA-3′ for MGMT, respectively.

### Cell cycle and cytotoxicity assay

For the cell cycle analysis, 10^5^ cells were trypsinized, washed with buffer solution and treated with 200 μg/ml RNAse A (Qiagen kit, Germany). Cells were fixed with 4% formalin and subsequently stained with 10 μg/ml propidium iodide (PI, Sigma Aldrich, UK) in the dark, as per manufacturer’s recommendations.

For the cell death estimation, the PI staining was also used to minimize variation between assays and validate the 3D cell death pattern as visualized with Draq7 (see below). 10^6^ cells were washed with buffer solution (same as in cell cycle assay) and resuspended in staining PI buffer solution, as per manufacturer’s recommendations. Supernatant material was collected to evaluate non-attached dead cells.

For both assays, samples were prepared and sorted with minimum incubation time. Cell flow cytometry was performed in FACSCalibur™ (BD Biosciences, USA) with excitation at 488 nm and analyzed using the FlowJo™ 10.8 (BD Life Sciences, USA) software.

### 2D drug-screening

In each well of a 48-well plate, 1 ml of a single-cell solution was seeded (50,000 cells/well) and cultured in standard lab conditions. After 24 h, cells were treated with a range of 5–2000 μM for TMZ (Sigma Aldrich, UK) and of 0.0015–5 μM for DOX (Sigma Aldrich, UK). Plates were incubated for 72 h. Medium’s phenol red has been washed before the MTT (Sigma Aldrich kit, UK) in vitro cytotoxicity assay was carried out, as per manufacturer’s recommendations. Sample absorbance was measured in a multi-well spectrophotometer (Lamda 2, Perkin Elmer, USA) at 590 nm. For each sample spectrophotometer reading, the mean of the two blank readings (negative control) was subtracted before any further calculation, in order to account for any background signal that can bias the MTT estimations. Positive control samples mean of untreated cells was also accounted for each of the experiments. Each treated condition counts at least two replicates per experiment and each experiment has been at least repeated twice.

### Spheroid generation and drug treatment

An initial single-cell suspension solution of approximately 625 cells /50ul of supplemented DMEM-F12 per well was used. Spheroids were generated in 96-well hanging drop plates (3D Biomatrix, USA). Spheroid growth was allowed for up to 3 weeks. On day 4 post-seeding, spheroids were treated with a range of concentrations of the anti-cancer agents TMZ (200–1000 μM) and DOX (0.0015–0.9 μM) as in^31^, either as monotherapy or in combination, based on the IC_50_ values previously estimated in 2D. Starting from 3 days post-treatment, half of the medium was replenished with fresh medium every two days. Images of the growing spheroids were captured in a Leica DFC310 FX inverted wide-field fluorescence microscope (Leica, Germany) using 4 × magnification in order to monitor the spheroid growth. Each condition counts at least 6 replicates per experiment and each experiment has been repeated at least 3 times. A schematic overview of the drug treatment experimental design can be found in supplementary Figure S1D.

### Fluorescence imaging

Light-sheet fluorescence microscopy (LSFM) imaging was used in order to visualize the intrinsic and drug-induced cell death of the multicellular spheroids over time, as in^43^. The spheroids were placed and stabilized inside fluorinated ethylene propylene (FEP) tubes (Bola, Germany) containing solidified Cygel (Biostatus, UK). The diameter of the FEP tubes was adjusted per spheroid diameter, varying between 800 and 1200 μm. The tubes were followingly inserted inside a tank made by anti-reflection optical glass (Hellma Analytics, Germany), filled with warmed water. Each spheroid was imaged sequentially at 4 orthogonal projections, which were then registered and fused together to form the final volumetric image with sub-cellular resolution. Maximum intensity projection (MIP) images were produced using Fiji^44^.

DOX penetration into the spheroids was determined by direct imaging of its autofluorescence (excitation: 488 nm, acquisition: 615/90 nm). The far-red nuclear dye Draq7 (Biostatus, UK) was used to label dead cells (excitation: 635 nm, acquisition: 650LP filter). Spheroids were treated with Draq7 16-20 h before imaging.

### Computational modeling

A probabilistic HDC mathematical model^45,46^ was developed to describe the drug mechanisms and effect on GB cells. In brief, the concentration of each drug was modelled as a continuous variable, while the GB cells were formulated as discrete variables living on a $h\times h$ lattice site which fits a single cell of fixed size (*h* = 15 μm), as in vitro previously estimated^42^. In the untreated condition, the GB cells can proliferate, become quiescent due to space competition or die due to spontaneous death. Upon treatment (Figure S7), cells can (i) pause division (a.k.a. mitosis M-checkpoint), (ii) enter a long-lasting quiescent period during the gap zero phase (G0); drug-induced cytostasis, (iii) die through necrosis; because of irreversible G0 (a.k.a. senescence) or drug-induced cytotoxicity. The states “Die” and “G0” were assumed as irreversible.

At the beginning of the simulation, each cell was randomly assigned an age, which corresponds to the cell cycle and increases. At each iteration period of the model, cells were able to divide when their age reaches their doubling time (τ = 22 h, as in vitro assessed^42^). A cell proliferates in case of available empty space on its surrounding 8 lattice cells (1-Moore neighborhood) and it can expand the searching nearby (up to 3-Moore neighborhood)^46^. If no space is available, it enters a reversible quiescent state. In order to avoid synchronization artifacts and account for the natural variability in the proliferative capacity of the cell population^42^, we introduce slight heterogeneity in the cell-cycle duration. Specifically, the doubling time is randomly selected from a normal distribution with a mean of τ = 22 h and a standard deviation of 1 h (see also supplementary Table S1). Dying cells first experience a lysis period, where they eventually degrade and are followingly treated as empty space.

Drug concentration was assumed a continuous variable in the model as regards diffusion, uptake and dilution^31^ and was described by a reaction–diffusion equation. Dirichlet boundary conditions were applied, in order to set a respective dose at the edge of the computational domain, thus mimicking the experimental conditions. We considered either spontaneous or drug-induced cell death. The main computational parameters that were assumed can be found in the Table S1.

The drug-induced cell fate decision was probabilistically made in each cell cycle during mitosis. The conventional in vitro dose–response curves were converted to a dose-dependent probabilistic cellular decision making of drug-induced cell death or cell-cycle arrest at the single-cell level. We assume that the probability of a cell to be affected by a given drug depends only on the local maximum extracellular drug concentration. The pharmacokinetics of cellular uptake and efflux pumps were not considered. Specifically, considering that a drug is administered at a specific dose, we assume $p$ the probability of a cell to remain unaffected by a specific drug concentration and proliferate, and $(1-p)$ the probability to be affected. We next assume $\lambda ,$ the probability of an affected cell to enter the G0 state, and $(1-\lambda )$ the probability of an affected cell to die. Thus, the probability of a cell to enter the G0 state equals to $\lambda (1-p)$ and the probability of a cell to die equals to $\left(1-\lambda \right)\left(1-p\right)$. The population of viable cells equals to the proliferating cells and those that have entered the G0 state. The formula that describes the progression of the viable population under drug treatment is shown in the supplementary file (Equation S1). If the untreated population follows exponential growth in ideal conditions, we can equate the theoretic ratio of treated to untreated viable population with the experimental dose–response curve. Yet, the system can be considered undetermined in the sense that various $(p,\lambda )$ probability pairs can produce the same inhibition effect. However, considering that DOX is predominantly cytotoxic, we can assume $\lambda =0$ and estimate the related dose-dependent probabilities. Similarly, as TMZ is predominantly cytostatic, we can respectively assume $\lambda =1$. We also computationally explore ranges between these two extreme scenarios. Note that if the drugs achieve the same inhibition level of the viable cell population at the end of the exposure period, the probability of a cell to enter the G0 state is different from the probability to die because cells in G0 state accumulate over time counting on the viable population.

In combination therapy, we assumed probabilistic independence for the drug-induced cell fate meaning that the probability of a cell to be affected by a drug does not change by the presence of the other drug (*null hypothesis*). Under that assumption, the combined probability describing the cell fate is derived from the respective probabilities in monotherapy. When both drugs are applied, a cell might be affected either by the first or the second drug only, or by both drugs (intersection of the probabilities) or even by none of them. Specifically, if ${P}_{A}$ is the probability of a cell to be affected by drug A (at a certain concentration) and similarly ${P}_{B}$ for drug B, then the probability of a cell to be affected by either drug A or B is equal to ${P}_{AB}={P}_{A}+{P}_{B}-P(A\cap B)$. Assuming independence, the probability of a cell to be affected by both drug A and drug B equals to $\left(A\cap B\right)=$ ${P}_{A} \cdot {P}_{B}$. In the case where the two drugs have the same effect on a cell, the application of the probability on cell fate under the presence of both drugs is more straightforward^47^. Yet, if drug A is cytotoxic and drug B is cytostatic, the cell fate at the intersection of the probabilities is unknown (see Figure S6). A variety of drug combinations were tested computationally under the null hypothesis. In that manner, beneficial doses, which minimize the drug doses while keeping the high efficacy, can be identified. The GB cells were placed on the lattice with two different initial configurations. The first configuration resembled the monolayer in vitro experiments, with low cellularity and homogeneously distributed drug. This configuration was used only to validate the correctness of the derived dose-dependent and drug-dependent probabilities. The second configuration was set to represent the planar central slice of the spheroids, with higher cell density and drug distribution described by a reaction–diffusion equation. In all presented simulations, the second configuration was used.

### Statistical analysis

Drug-response was evaluated measuring spheroids area reduction as opposed to control spheroids in regular intervals, for up to 3 weeks. Spheroid area was segmented using Fiji^44^. The growth curves were analyzed using GraphPad Prism 9.3.1 (GraphPad Software, Inc., USA) with regression analysis.

The dose–response curves reflect the number of viable cells for the treated condition relative to the untreated condition viable population and were generated using the formula:

1 $$\% growth\,inhibition = \left( {\left( {{\text{positive}}\,{\text{control}} - {\text{test}}\,{\text{value}}} \right)*100} \right)/{\text{positive}}\,{\text{control}}$$

of the 2D MTT measurements and the 3D area spheroid values for the day 10 after treatment as opposed to the log[c] of the relevant drug concentrations. The IC_50_ was defined as the drug concentration where half of the cell population was inhibited.

The mean value of the root mean square error (RMSE) over all timepoints as compared to the untreated condition was used to estimate the accuracy between the in silico and the in vitro results under the formula:

2 $$RMSE = \sqrt {\mathop \sum \limits_{t = 1}^{{N_{t} }} \frac{{\left( {Inhib\_area_{v} \left( t \right) - Inhib\_area_{s} \left( t \right)} \right)^{2} }}{{N_{t} }}}$$

where Inhib_area_v_ refers to the in vitro area in treated spheroids divided by the untreated, Inhib_area_s_ refers similarly to the in silico tumor areas, and N_t_ refers to the number of timepoints.

## Results

### GB cells pathophysiological profiling shows potential resistance to Temozolomide

As it can be seen in Fig. 1B, the histopathological estimation of the patient’s biopsy shows that the neoplastic cells are glial fibrillary protein (GFAP) positive suggesting poor survival, and they have a proliferation index (Ki67) around 25%. Sample was collected during the gross maximal safe resection of the primary GB tumor (Fig. 1A). The RNA levels of the MGMT that often correlates to TMZ resistance were also examined for both U87MG and the GBP08-P0. As expected, the U87MG cells express very low levels of MGMT and they are therefore considered TMZ-sensitive^1^. Similarly, the GBP08-P0 cells have negligible MGMT expression (Figure S1A).

As it can be seen in supplementary Figure S1B and C, a rather equal distribution between cell cycle phases has been depicted for the GBP08-P0 cells, unlike U87MG where most cells seemed to be on G0-G1 cell cycle phase. Furthermore, intrinsic cell death is found to be <  < 10% for both cell lines, in line with the bibliographic values for the spontaneous cell death in gliomas are 5–11%^48^.

### Doxorubicin can inhibit GB growth alone or in combination with Temozolomide

**Figure 2:**
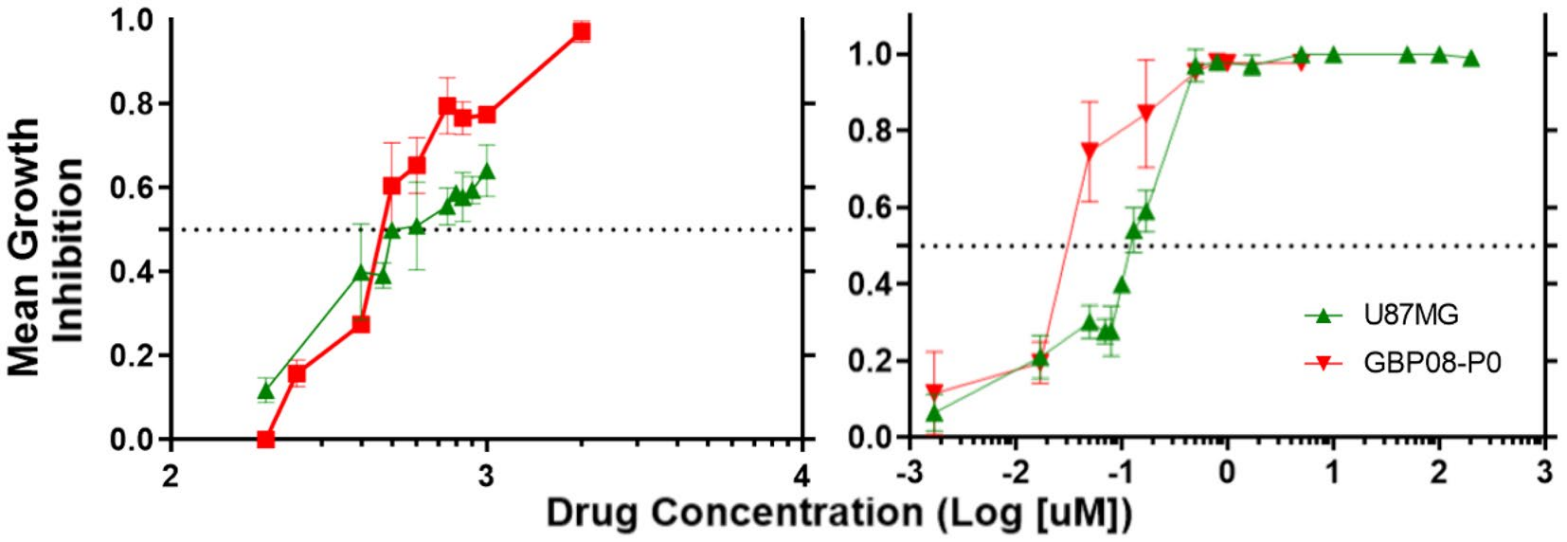
2D drug-response. Temozolomide (right) and Doxorubicin (left) growth inhibition plots for the U87MG (green) and the GBP08-P0 (red) cells, respectively. Equation (1) was used as described in Methods.

Figure 2 summarizes the drug-response curves of the 2D in vitro drug-screening for the U87MG and GBP08-P0 GB cell lines. Both cell lines appear to have similar response against TMZ, where half population inhibition is achieved only in drug concentrations over 500 μM (in line with the reported estimates for 72 h^49^). Unlike TMZ, DOX is effective in four orders of magnitude lower concentration, and GBP08-P0 cells (IC_50, DOX_≈ 0.05 μM) are more sensitive than the U87MG (IC_50, DOX_≈ 0.13 μM).

**Figure 3:**
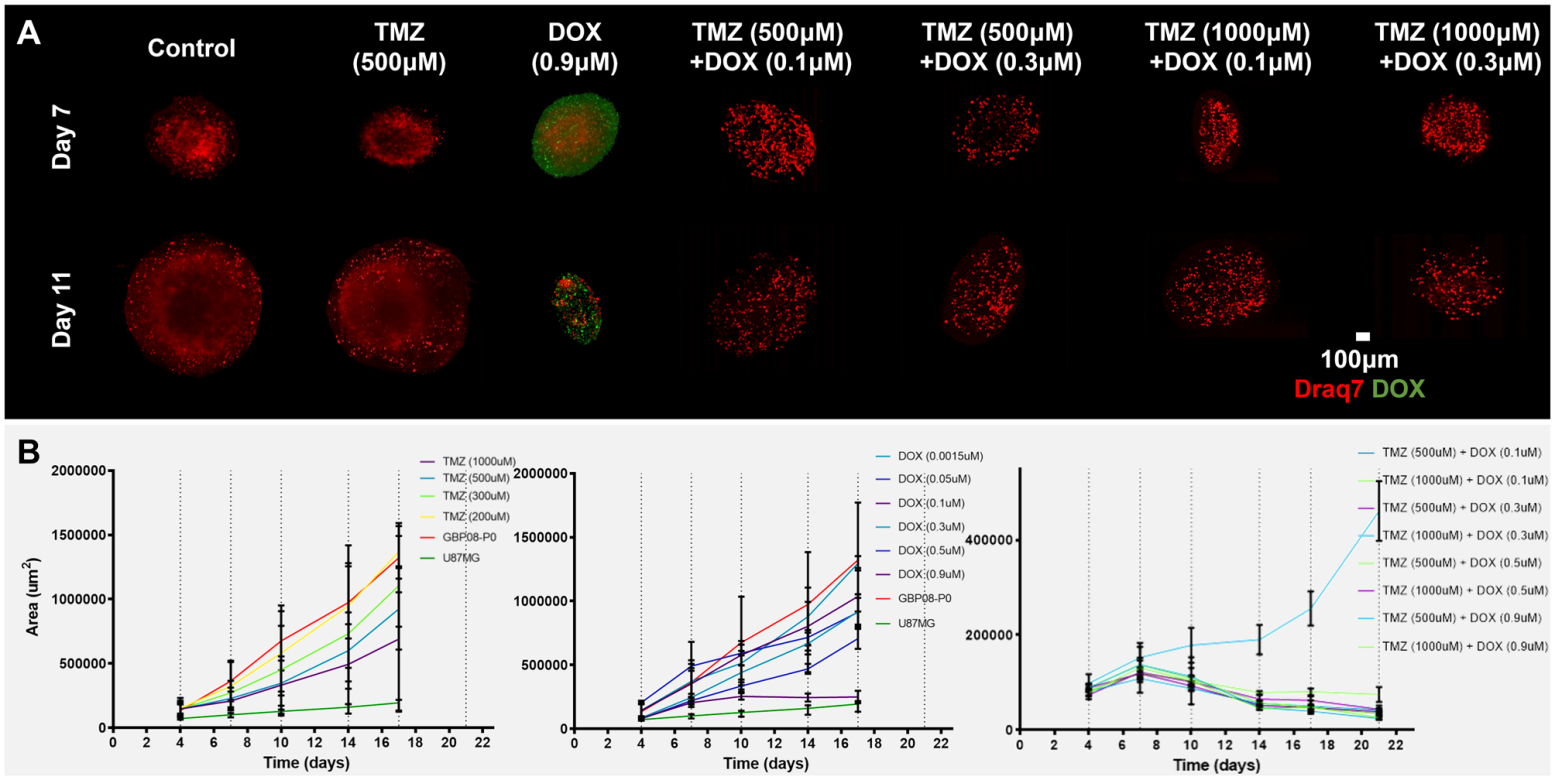
3D drug-response. (**A**) Reconstructed LSFM MIP images labeled for cell death (Draq7, shown in red). Representative GBP08-P0 primary spheroids are depicted for 3- and 7-days post-treatment (depicted as Day 7 and Day 11 post-seeding, respectively) for either control, monotherapy or combination therapy. Note the high imaging depths achieved inside the large control and TMZ-only treated spheroids. DOX autofluorescence (green) can be seen in the DOX-treated spheroids, where the concentration is higher than 0.9 μM in order to be detected. Spheroids of 1000 μM TMZ treatment are similar to 500 μM, and 0.1–0.3 μM DOX-treated spheroids are similar to control for the timepoints shown here. Scale bar is set to 100 microns. (**B**) Dose–response curves for TMZ (left), DOX (middle) and combination therapy (right). Dose–response curves refer only to GBP08-P0 spheroids. U87MG (green) and GBP08-P0 (red) untreated cells are used as a control between conditions. Notice that almost all combination therapy doses lead to irreversible growth inhibition.

Although the U87MG cells 2D drug-response curves are not that different to the GBP08-P0 cells, this is not the case for the spheroids’ treatment (see also supplementary Figure S2), where higher dose of DOX should be used in order for the cell death to be triggered. Additionally, as it can be seen in Fig. 3B, U87MG indicative spheroid growth over time is very slow, even in drug absence. Based on the histological and molecular profile, as well as the drug responsiveness and growth pattern, the U87MG cells have not been further used for drug combination experiments in either the in vitro or the in silico experiments, and they are only used as a reference broadly-used GB cell line. In the following, only results focusing on the GBP08-P0 cell line observations are shown and more information on the U87MG cells can be found on the supplementary material (Figures S1-S3).

TMZ-treated spheroids exhibit a dose–response pattern, but all drug concentrations tested are able only to suspend the overall growth. As it can be observed already from the monolayer cultures of Fig. 2, in Fig. 3A,B, even at 500 μM of TMZ, spheroids overall growth and death pattern resembles the one of the untreated spheroids. Unlike TMZ, DOX is effective in very low doses, even four orders of magnitude less. More specifically, DOX-treated spheroids with drug concentration > 0.5 μM are irreversibly growth-inhibited throughout the period of study. Since there is no differential pattern of the induced-cell death between control and TMZ-treated spheroids, it can be assumed that TMZ homogeneously affects all spheroid regions. This assumption is in line with the theoretical high TMZ distribution^50^. Unlike TMZ, DOX autofluorescence is higher in the spheroid periphery, in line with its known accumulation in the outer tumor layers^51^.

As regards the combination therapy study, all drug combinations resulted in greater inhibition than the respective drug concentrations tested separately. From all the conditions, only 500 μM TMZ + 0.1 μM DOX results in reversible growth inhibition, yet only after 12 days post-treatment. The death pattern of all combination-treated spheroids is similar up to day 3 post-treatment, as shown in the Draq7-labeled nuclei in Fig. 3A. However, as it can be seen from the growth inhibition curves in Fig. 3B, growth inhibition is observed 3 days post-treatment, possibly indicating cumulative necrosis that could be caused by the combination of the two drugs (also observed as debris surrounding the spheroid tumors in supplementary Figure S1E).

### The in silico predictions align with in vitro results for monotherapy

The spatiotemporal response of the GB spheroids to each drug compound is formulated, calibrated and validated against the in vitro data for monotherapy. At first, DOX is assumed to have a predominantly cytotoxic effect and TMZ a mainly cytostatic. Then ranges between these extreme assumptions are considered. At last, the response of GB spheroids in combination therapy is predicted (null model). Beneficial doses of interest are tested experimentally and compared with the null model.

**Figure 4:**
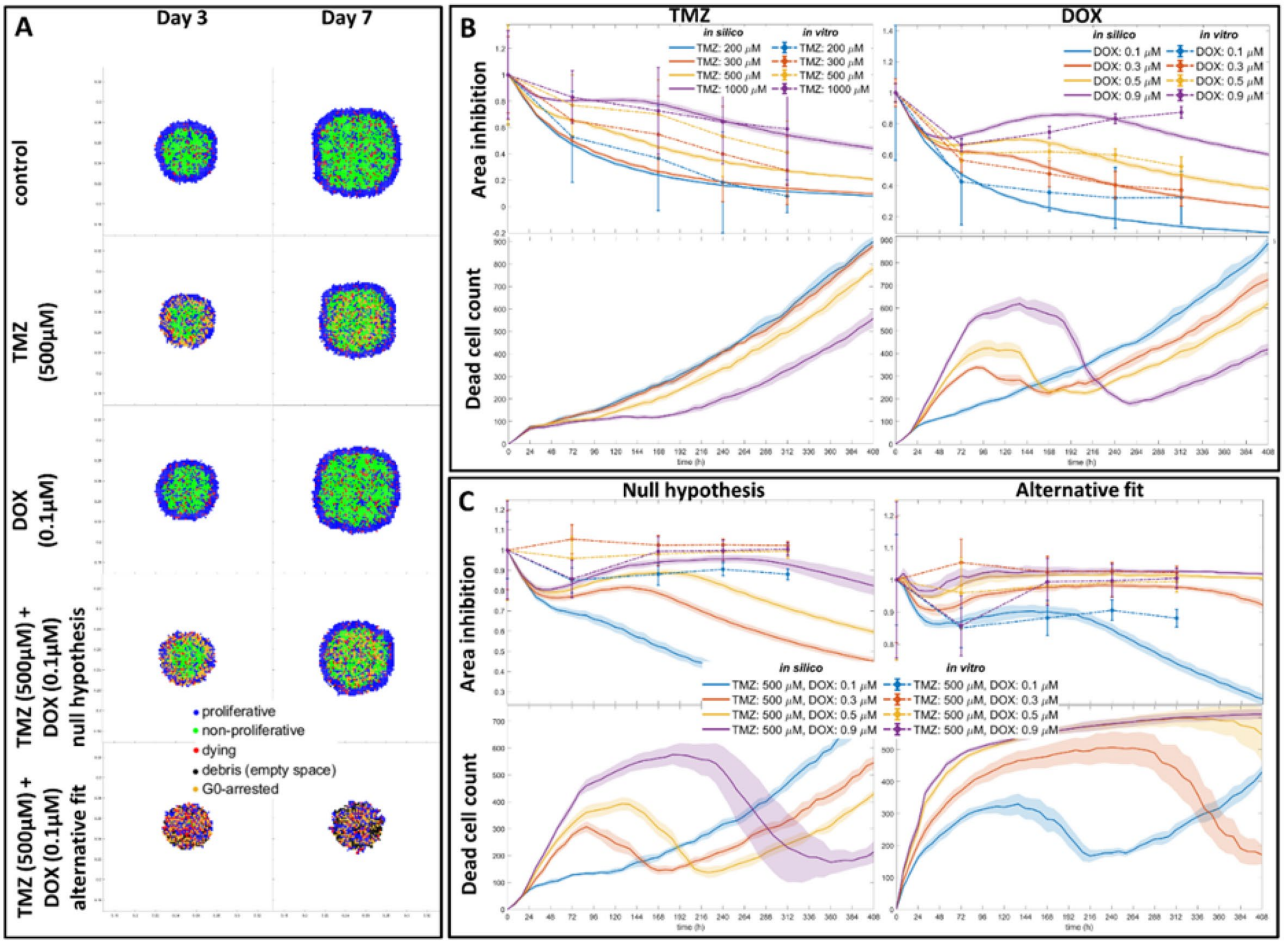
Simulated drug-response. (**A**) Spatial distribution of cells for 3- and 7-days post-treatment for the control, TMZ, DOX and combination representative concentrations. Both the null hypothesis and the alternative fit simulations are shown for the combination therapy. Proliferative (blue), non-proliferative (green), dying (red), G0-arrested (yellow) cell states and debris (black) are depicted. (**B**) Monotherapy in silico–in vitro temporal evolution of growth inhibition (as opposed to the untreated condition) and cell death. Intrinsic cell death rate and lysis period are the same. There is long-term assumed probability for DOX-induced cell death. Drug uptake and diffusion are assumed as γ***tmz*** = 1.4 × 10^−10^ M/(cell s), *Dtmz* = 8.68*e* − 07cm^2^/s for TMZ and γ***d***ox = 1.4 × 10^−13^ M/(cell s), *Ddox* = 8.68*e* − 08cm^2^/s for DOX, respectively. (**C**) Combination therapy in silico–in vitro temporal evolution of growth inhibition (as opposed to the untreated condition) and cell death. For the null hypothesis, drug uptake and diffusion are assumed as in the respective monotherapy in B. For the alternative fit hypothesis, no diffusion coefficient and uptake are considered, while DOX is diluted by 2/3 every other day after 72 h.

Each computational experiment has been repeated 10 times. An extensive parameter study can be found in the supplementary file. Results of 3- and 7-days post-treatment are shown in Fig. 4A. In Fig. 4B, the overall simulated growth inhibition for a range of doses for each drug is compared to the respective biological results. Interestingly, a solid tumor evolves differently, due to spatial gradients and space competition, in the presence of a cytostatic drug relative to the presence of a cytotoxic drug, which can be also seen in the respective cell death curves. Unlike the cytostatic drug, cell death peaks in the initial timepoints proportionally to the cytotoxic drug dose and before the drug is considerably diluted, nonetheless, intrinsic cell death affects more spheroids of larger size in the later timepoints. The results for different ranges between the two extreme scenarios are presented in the supplementary material, considering that: (i) various probability pairs (p,λ) can produce the same viability ratio in the 2D assay, (ii) it is expected that neither of the two drugs is purely cytostatic or cytotoxic, and (iii) this effect is likely cell line-dependent, dose-dependent, condition-dependent (hypoxia, etc.), as well as time-dependent.

### The in silico predictions diverge from the drug-response if Temozolomide-Doxorubicin independence is considered

A range of DOX doses was followingly tested against various concentrations of TMZ. The combined effect was estimated based on the combined probabilities, as described in Methods, following the parametrization that was indicated by the respective monotherapy simulations (null model). In Fig. 4C, the overall in silico growth inhibition and the cell death of the combination therapy is compared against the respective in vitro growth curves over time. In Table 1, the HDC model’s performance underscores all the combinations tested and predicts relapse. Under null hypothesis rejection, a set of parameters were also tested to better approximate the in vitro results (see also supplementary Figures S9-S13). In Fig. 4C, the alternative fit predictions are shown under the assumption that there is minimal dilution and no diffusion for DOX (see also Table 1). Interestingly, the cell death curves of the alternative fit predictions resemble more the DOX-only case in Fig. 4B, while the percentage of proliferative cells is minimized from the early timepoints as it can be seen in Fig. 4A. Note that when the assumptions regarding the cytotoxic-cytostatic effect varied, the conclusion regarding the in vitro–in silico discrepancy of combination treatment remained unaffected (see also supplementary material).

**Table 1:** The RMSE scores are shown for all monotherapy and combination therapy dose ranges.

TMZ		DOX		TMZ + DOX		
Concentration (μM)	RMSE	Concentration (μM)	RMSE	Concentration (μM)	Null hypothesis RMSE	Alternative fit RMSE
200	0.0723	0.1	0.1275	500 + 0.1	0.4195	0.0780
300	0.2043	0.3	0.0397	500 + 0.3	0.3514	0.0756
500	0.1803	0.5	0.0520	500 + 0.5	0.1861	0.0267
100	0.0380	0.9	0.0995	500 + 0.9	0.0521	0.0808

## Discussion

In this study, a GB predictive HDC model was used in order to explain the response effect of DOX-treatment as monotherapy and in combination with TMZ. Although TMZ responsiveness was found to be relatively low, in line with the metronomic chemotherapy principles, several drug combination concentrations, which also keep the efficacy high, were explored. In vitro avascular tumor growth was longitudinally monitored (instead of end-point experiments) for patient-derived (instead of secondary) GB spheroids (instead of 2D cultures). Interestingly, the TMZ-DOX drug combinations tested in vitro: i) were beneficial as compared to the respective monotherapies, and ii) were able to lead to irreversible growth inhibition for up to 3 weeks post-treatment. Based on our computational results, the TMZ-DOX therapeutic scheme can act directly on the GB proliferative cells and lead to overall spheroid growth inhibition with a dual mechanism of action; first, growth arrest and subsequent cell death because of either irreversible G0 phase or necrosis. Simulated results were validated based on the respective biological drug-screening data and the predictive competence of the model was underscored under no synergy assumption, therefore, alternative mechanisms were explored.

The conventional secondary cell lines are limited in recapitulating the pathophysiologic complexity of high-grade brain cancer. From several own-established patient-derived GB cell lines tested^42^, GBP08-P0 was found to fulfil the following criteria: (i) desired MGMT status as compared to the U87MG cells that are considered deficient for MGMT^52^, (ii) relative correlation between 2 and 3D in vitro drug-screening, and (iii) zero animal passaging needed during the primary cell line establishment in order to achieve the highest correlation to the patient original responsiveness. Interestingly, the difference in the overall spheroid growth between the U87MG and the GBP08-P0 cells cannot be attributed solely to the average doubling time differences^42^, rather than the differential responsiveness to the two drugs as the U87MG are a lot more resistant to DOX and most TMZ tested doses are not much different to control growth. TMZ recommended dosage for the cells varies between 10-1000uM or even more depending on the cell line, the cell culture (2D or 3D) and the cytotoxicity assay used^1^, for the animals is 120 mg/kg/day^1^ and for the patients is 150–200 mg/m^2^^8,53^. In this study, TMZ concentrations over 500 µM were found to be effective, which is outside the range of optimal plasma level (30–80 µM) achieved during oral chemotherapy^54^. Though the maximum recommended cumulative dose of DOX for patients is 450–550 mg/m^2^^55^, it has been shown that the GB cells effective concentration is extremely low ~ 10–50 ng/ml^18^ or ~ 0.05uM in 2D and 0.17uM in 3D in vitro studies^56^, which was confirmed by our results. Alternative routes of drug administration, such as molecule modifications, local drug administration during surgery or implanted pumps in development, are expected to enhance local drug concentration to compensate for poor responsiveness and need for higher dosing.

In order to parametrize the HDC model, we focused on the main mechanisms of action reported for the two drugs^57^, the drug serial dilutions of the biological experiments, and the spatial distribution of the drugs within the spheroids. Methodologically, the combination effect of drugs can be studied with respect to effect-based strategies (i.e. Bliss independence)^58^ and dose–effect-based strategies with Loewe additivity (a.k.a. dose additivity)^59,60^ and the related isobologram analysis. However, these models, when applied to the fraction of cells affected, overlook the probabilistic nature of cell events induced by drug exposure. By delving into the intricate complexities of drug-induced cell death and inhibition of cell division, we can improve null models, resulting in more accurate estimates of drug efficacy and combination effectiveness^47^.

Our proposed approach provides a suggestion on the dual TMZ-DOX mechanism of action that has not yet been effectively described counting on a formulation, calibration and validation of a spatiotemporal response model of tumors to monotherapy.

A computational parameter study was performed in which: (1) limited dilution was assumed for the cytotoxic agent (sustained death probability simulating DOX being re-released after cell death^17^, as it can also be seen by DOX retention in Fig. 3A), (2) various drug penetration was considered, (3) the cell fate at the intersection of the probabilities and (4) the cytotoxic-cytostatic assumption for DOX and TMZ were also reconsidered. None of these alternatives was capable to well resemble both monotherapy and combination experiments. Tumor dormancy without relapse was in silico predicted and in vitro validated only after rejecting the null model in which the probabilistic drug independence may hold, yet environmental factors differ when the drugs act alone or in combination. To this end, the assumption that DOX penetration is different when administered alone or in combination with TMZ, led the HDC model to better resemble the in vitro observations.

It is important to note that, in our study, the presumed time-invariant probabilities linked solely to external drug concentration may undergo changes over time due to dynamic pharmacokinetics. In our future studies, we aim to incorporate temporal probabilities affected by pharmacokinetic processes. These refined time-dependent probabilities will subsequently inform combination treatments. As our comprehension of the spatiotemporal dynamics of monotherapy deepens, the null model's robustness will increase.

Widely used null models, such as the Bliss independence, applied to our experimental data (Figure S15), also provide support for interaction between the two drugs. This observation suggests the presence of a potential different mechanism of action when TMZ and DOX are combined in drug concentrations that do not cause lethal toxicity. However, no pharmacodynamic/pharmacokinetic molecular interaction has been reported between the two compounds (except for artificial cross-linkage^61^), therefore there is no implicit drug molecular synergy that can be presumed. The in vitro–in silico paradox observed in our experiments can be biologically explained by the changes in the inter- and intra-cellular tumor microenvironment caused by either of the two regimens and affecting the acute mechanism of action, as well as the indirect clonal cell response to either of the two agents or both, in line with the multidrug resistance phenotypes. An example of such an environmental change could be the alternate inhibition of the p-glycoprotein ATPase that was recently reported in different GB cell lines treated with TMZ-DOX^62^. Additionally, metronomic TMZ treatment can lead to permanent cell cycle arrest and eventually, cytotoxicity^63^. The typical damage repair time of the tumor microenvironment in order to compensate for drug-induced toxicity is in the order of a few hours^53^, yet in our computational approach we did not account for the effect of one drug altering the effect of the other (memory effect). Such observations, further highlight the importance of computational predictive algorithms in the experimental design in order to guide a biologically challenging study and vice versa, the validation of the computational biology with real data in order to better elucidate GB physiology^64^. We will further investigate multidrug phenotype and effective timing response in our future studies. In the future, we also aim to delve into the dynamics during and post-treatment and incorporate the pharmacokinetics and pharmacodynamics of the drugs under consideration. This iterative approach will further refine our understanding of monotherapy and combination simulations. Furthermore, drug optimization strategies will be explored computationally and tested against commonly used therapeutic regimens for patients that involve repeated cycles of drugs. Nonetheless, this framework can be extended to account for additional chemotherapeutic agents other than TMZ and DOX or alternative treatment strategies, such as radiation.

Although LSFM imaging enabled monitoring of the avascular tumoroids for an unparalleled time period, no quantification of the necrotic spheroid areas was enabled because we were limited down by the spheroid size. Further on, the LSFM probe used neither labels cell arrest nor cell death types. Our results demonstrate the potential of this technology to quantitatively assess the distribution, drug penetration and cytotoxic potency of anti-neoplastic agents in living 3D cell cultures and to serve as a useful tool in further exploring the complicated mechanisms highlighted in TMZ-DOX combination.

It has been shown that the TMZ efficacy is essentially reduced under hypoxic conditions when tested for the U251N GB cell line^65^. Also, in a recent study^66^, the inner hypoxia-preserved GB cells were more TMZ-chemoresistant and this was MGMT-related since hypoxia-induced factors, such as HIF1-α, may alter the MGMT phenotype. Hence, in future experiments, chemically-induced hypoxia should be also tested.

## Conclusions

Our computational approach, based on the 2D derived dose–response curves of each drug and the 3D longitudinal experiments, was able to reveal a supra-additive TMZ-DOX response.

Either by designing drug delivery systems inserted in a brain tumor resection cavity or new forms of chemical compounds, it seems that DOX can be enrolled in brain cancer therapy in order to amplify response outcome, as regards both the timing and the end target of the treatment. We speculate that the experimental framework described here can be extrapolated in order to account for variable drug responsiveness for the two regimens of other GB cell lines provided that the in vitro experimental data can be acquired. *To our knowledge, this is the first time a computational predictive algorithm is used to predict the TMZ-DOX effect in* GB *enabling a drug-screening tool that is difficult to be experimentally tested and challenging to be clinically applied.*

Given the aggressiveness of GB symptomatology and the excessive need for fast-decision theranostic strategies, we believe that our suggested work can serve as a clinical tool towards precision medicine that is currently limited to molecular GB subtypes and rather doubtful biomarkers, further promoting patient’s quality of life.

### Supplementary Information

Supplementary Information.

## Data Availability

The datasets and code used and/or analysed during the current study are available from the corresponding author on reasonable request.

## Abbreviations

BBB: Blood brain barrier
DOX: Doxorubicin
FEP: Fluorinated ethylene propylene
GB: Glioblastoma
HDC: Hybrid discrete–continuous
LSFM: Light-sheet fluorescence microscopy
LP: Low pass
MGMT: Methyltransferase
TMZ: Temozolomide
