# Supplementary material for: The Temozolomide–Doxorubicin paradox in Glioblastoma in vitro–in silico preclinical drug-screening
Source: Sci Rep. 2024 Feb 14;14:3759. doi: 10.1038/s41598-024-53684-y (PMC10866941; doi:10.1038/s41598-024-53684-y)
Supplement: Supplementary file 1 — Supplementary Information. [file 41598_2024_53684_MOESM1_ESM.docx]

The Temozolomide-Doxorubicin paradox in Glioblastoma

*in vitro-in silico* preclinical drug-screening

Mariam-Eleni Oraiopoulou ^1,2^, Eleftheria Tzamali ^1^, Stylianos E. Psycharakis ^3,4^, Georgios Tzedakis ^1^, Takis Makatounakis ^5^, Katina Manolitsi ^6^, Elias Drakos ^4,6^, Antonis F. Vakis ^4,6^, Giannis Zacharakis ^3^, Joseph Papamatheakis ^5,7^, and Vangelis Sakkalis ^1,*^

**^1^** Institute of Computer Science (ICS), Foundation for Research and Technology-Hellas (FORTH), Greece

**^2^** Cancer Research UK – Cambridge Institute, University of Cambridge, United Kingdom

**^3^** Institute of Electronic Structure and Laser (IESL), Foundation for Research and Technology Hellas (FORTH), Greece

**^4^** School of Medicine, University of Crete, Greece

**^5^** Institute of Molecular Biology and Biotechnology (IMBB), Foundation for Research and Technology Hellas (FORTH), Greece

**^6^** University General Hospital of Heraklion (PAGNI), Greece

**^7^** Department of Biology, University of Crete, Greece

* Corresponding author: Vangelis Sakkalis ([sakkalis@ics.forth.gr](mailto:sakkalis@ics.forth.gr))

**Supporting Information Text**

***In vitro-in silico* drug-response study**

We aim to provide a mathematical description of how *in vitro* spheroids evolve and respond to chemotherapeutic treatment. A simplistic description is provided, which however accounts for the most relevant underlying biological processes and which could be calibrated (at some extent) by the experimental data. Unconstrained parameters, which are not experimentally measured, are explored within the mathematical framework for their impact on spheroid dynamics.

We utilized parameter values measured in 2D *in vitro* cultures (i.e. proliferation rate, spontaneous death rate, drug response). The dose-response curve for each drug is converted to a dose-dependent probability for an event to occur (detailed description is provided in the following section). We computationally developed a spatiotemporal model for predicting drug responses in monotherapy (DOX, TMZ). A hybrid discrete-continuous mathematical approach is adopted. To resemble the 3D growth, the model accounts for diffusion gradients and space competition (mechanical cell contact inhibition) among cells. We calibrated the proliferation rate and the depth of the proliferative rim to fit the untreated (control) spheroid expansion. These parameters were kept fixed in all the simulations thereafter. The drug gradient was then calibrated in order for the model predictions to fit the experimental spheroid drug-response curves (Figure S3). We assumed cellular properties like intrinsic cell death rate and lysis period the same for all cancer populations. Note that in our simplified modeling setup, the drug pharmacokinetics (i.e. drug accumulation within the cell, drug binding, and drug metabolism) are not explicitly considered (as for example in 1, 2, 3). Therefore, the drug-induced cell fate depends on the external drug concentration. In that manner, by imposing drug gradients in the system, we directly alter the drug-induced effect on the cell.

Once the model is calibrated in the 3D geometry for monotherapy, it is used to describe combination therapy under the drug independence (no-interaction) assumption as a null model (see description in the following section). The combination probabilities are applied, and all other parameters are kept the same as in monotherapy (null model). Various concentrations of drug A can be tested against various concentrations of drug B estimating their combined effect under the probabilistic drug independence assumption (Figure S6).

Critical parameters that may affect the spheroid evolution and treatment response including the initial spheroid size (Figure S8), lysis period (Figure S9), latency period (Figure S10), and drug diffusion/penetration (Figure S11) are further explored. At first, we considered DOX as a purely cytotoxic drug and TMZ as a purely cytostatic drug. We then relaxed this hypothesis to explore how changes in this hard assumption affect the spheroid evolution (Figure S12). We also tested different dilution rates of the drug that indirectly regulate the post-treatment probabilities (Figure S13). The impact of the intersection probability in combination therapy is also explored (Figure S14).

The proposed computational model predicts the 3D *in vitro* experiments only after rejecting the null model meaning that conditions and assumptions applied in monotherapy are different when the two drugs are combined. Assuming different penetration of DOX in combination therapy relative to monotherapy (sensitizing in that manner the cells to the treatment), the simulations well-resemble the experimental curves of combination treatment.

A hybrid discrete-continuous model is assumed, in which individual cancer cells are described by cellular automata, and the drug concentration is considered a continuous variable. We use a 2D fixed regular lattice with a size of L = 0.5 cm to represent a planar slice through the 3D tumor spheroid, defining our computational domain. Cancer cells are treated as distinct entities residing on the 2D lattice. Each lattice site (*h x h*) can accommodate only one cancer cell. In this context, cells follow a set of rules, allowing them to proliferate, push neighboring cells while in mechanical contact, become quiescent due to contact inhibition, undergo spontaneous death, lysis, and experience drug-induced death. The spatiotemporal evolution of the drug concentration, $d$ is described by a reaction-diffusion equation (i.e. $\partial d/\partial t=\nabla\cdot(D_{d}\nabla d)-\gamma_{d}c_{i,j}d$) in which $D_{d}$ corresponds to the diffusion coefficient of the drug within the spheroid, and the term $\gamma_{d}$ corresponds to uptake rate of the drug by the cell. The term $c_{i,j}=\{0,1\}$ expresses whether there is a cell at position $\left( i,j \right)$ of the lattice. The drug concentration is rescaled with the maximum drug concentration administered in each experiment. In the non-dimensional description, the concentration of the drug at the edge of the computation domain was set to one through the application of *Dirichlet* boundary conditions. The non-dimensional value of one is properly readjusted to correspond to different drug concentration in each experimental setup.

For all the simulations performed in this work, the commercial software MATLAB was used. The numerical solution of the PDEs has been developed in C language.

***In silico* drug-response study**

**Translate the 2D dose-response curve into a probabilistic cellular decision making**

The widely used endpoint 2D *in vitro* assay used in our experiments (MTT assay), quantifies the number of viable cells relative to the untreated population growth providing relative growth inhibition information, over a wide range of drug concentrations. The measurements were performed at the end of the treatment period, which in our experiments corresponds to 72 hours.

We assumed that both drugs act during mitosis. We assumed that the cell population is in the exponential phase for the duration of the 2D experiments. We assume that the drug-induced phenotypic events (i.e. cell death or cell cycle-arrest) occurring in a cell population can be described by a homogeneous *Poisson* process in which the long-term average event rate is constant. Assuming a *Poisson* process, the event rate reflects the probability with which such events occur in individual cells within short time intervals. Under that respect, the mean values of the dose-response curves for each drug are converted to a dose-dependent probability for an event to occur.

Specifically, we assume *p* the probability of a cell to remain unaffected by a specific drug concentration and proliferate. Thus, *(1-p)* is the probability of a cell to be affected by the drug. We assume *λ* the probability of an affected cell to undergo mitotic arrest (pause division) and *(1-λ)* the probability of an affected cell to die. Then, the probability of a cell to pause division equals to *λ(1-p)* and the probability of a cell to die equals to *(1-p)(1-λ)*. Note that over time, the population of live cells equals to the proliferating cells and the cells that have undergo mitotic arrest, which accumulate over time. The probabilities of the drug-induced phenotypic events are assumed constant.

Discretizing the cell processes in time, we assume *N_o_* an initial cell population. Let *N_k_* be the current cell population at time step *k* (note that *k=t/τ*, where τ equals to cell’s doubling time), *P_k_* be the percentage of live cells that proliferate, and *G_k_* represent the percentage of cells that undergo mitotic arrest at the time step *k*. The live population at a particular time step *k* equals to:

${Live}_{k}=\sum_{n=0}^{k} G_{n}^{L}+ P_{k}=2\lambda\left( 1-p \right)N_{o}\frac{1-\left( 2p \right)^{k}}{1-2p}+\left( 2p \right)^{k}N_{o}, for p\neq\frac{1}{2}$ Equation S1

${Live}_{k}=\sum_{n=0}^{k} G_{n}^{L}+ P_{k}= 2\lambda\left( 1-p \right)N_{o}k+\left( 2p \right)^{k}N_{o}, for p=\frac{1}{2}$ Equation S2

Assuming a free, untreated growth, the population follows exponential growth that is ${Live}_{free}^{k}=N_{o}2^{k}$.

The cell viability curve at time *k* is then given by $S_{k}=\frac{{Live}_{k}}{{Live}_{free}^{k}}$.

We assume that the probabilities *p, λ* depend on the external drug concentration; they are applied every time the cell attempts to proliferate, and they are determined by the dose-response *in vitro* monolayer measurements, which are estimates of *S_k_*. As the system can be considered undetermined, meaning that various (*p,λ*) probability pairs can produce the same effect, we will firstly assume two extreme scenarios; a purely cytotoxic drug, where *λ* is minimized (practically *λ* =0) and a purely cytostatic drug, where *λ* is maximized (practically *λ* =1). Later, we will relax this assumption. Note that in the absence of random death, if *p* equals to zero in a purely cytostatic drug, meaning that all cells undergo mitotic arrest, the cell population remains constant over time. Nevertheless, the cell viability decreases, reaching zero at long exposure times as the untreated population exponentially grows decreasing their ratio between treated and untreated populations.

We have assumed that cells, which undergo drug-induced mitotic arrest, remain in that state throughout the course of the experiment, implying permanent cell failure to resume cell division upon release from the drug or drug consumption/ degradation. Furthermore, prolonged mitotic arrest prior to cell death has not been considered in this framework.

**Null hypothesis for combination therapy**

When both drugs are applied simultaneously in the population, a cell might be affected either by the first drug only, or by the second drug only, or by both drugs (intersection of the probabilities) or even by none of them continuing its proliferation. Figure S6B depicts the main cell states, as well as the two extreme outcomes regarding the cell fate that may occur when a cell is affected by both drugs. If at the intersection drug A dominates, the combination probabilities follow the left tree, whereas under the dominance of drug B, the right tree is followed. We investigated how the cell fate at the intersection affects the growth dynamics. Various concentrations of drug A were tested against various concentrations of drug B estimating their combined effect under the probabilistic drug independence assumption.

Specifically, if $P_{A}$ is the probability of a cell to be affected by drug A (at a certain concentration) and similarly $P_{B}$ for drug B, then the probability of a cell to be affected by either drug A or drug B is equal to $P_{AB}=P_{A}+P_{B}-P(A\cap B)$. Note that these probabilities are defined the opposite way here compared to how we have defined them in the previous paragraph. Assuming independence, the probability of a cell to be affected by both drug A and drug B equals to $\left( A\cap B \right)=$ $P_{A} \cdot P_{B}$. In the case where the two drugs have the same effect on a cell, i.e. cause cell division arrest or cytotoxicity, the application of the probability on cell fate under the presence of both drugs is more straightforward. Yet, if drug A is cytotoxic and drug B is cytostatic, the cell fate at the intersection of the probabilities is unknown. The two extreme outcomes regarding the cell fate that may occur when a cell is affected by both drugs are illustrated in the sequential drug application scheme. If at the intersection drug A dominates, the combination probabilities follow the left tree, whereas under the dominance of drug B, the right tree is followed. Reality can be anywhere between these extremes. Unless stated otherwise, the simultaneous drug application is used in our simulations with φ, the probability at the intersection equal to 0.5. However, we have also investigated how the cell fate at the intersection affects the growth dynamics and the overall response to treatment.

**Simultaneous scheme:**

$P\left( affected by A \right)=P_{A}-\left( \varphi-1 \right)P\left( A\cap B \right)=P_{A}-\left( \varphi-1 \right)P_{A}P_{B}$

$P\left( affected by B \right)=P_{B}-\varphi P\left( A\cap B \right)=P_{A}-\varphi P_{A}P_{B}$

If at the intersection Drug A dominates, then φ=1:

$P\left( affected by A \right)=P_{A}$ and $P\left( affected by B \right)=P_{B}-P_{A}P_{B}=P_{B}(1-P_{A})$

Similarly, if at the intersection Drug B dominates, then φ=0: $P\left( affected by B \right)=P_{B}$ and $P\left( affected by A \right)=P_{A}-P_{A}P_{B}= P_{A}(1-P_{B})$

**Sequential scheme (left tree):**

$P\left( affected by A \right)=P_{A}$ and $P\left( affected by B \right)={P_{B}(1-P}_{A})$

**Sequential scheme (right tree):**

$P\left( affected by B \right)=P_{B}$ and $P\left( affected by A \right)={P_{A}(1-P}_{B})$

**Simulating the spatiotemporal response to treatment**

2D experiments can provide us with the pure probabilities of drug effect on cells during the exposure time. Yet, to further predict 3D tumor evolution, spatial competition among cells has to be considered (most cells are quiescent in this arrangement due to cell contact inhibition), drug penetration and post treatment dynamics which are highly unknown and challenging to be experimentally assessed. As already mentioned, we assume that the drug-induced event probabilities applied every time the cell undergoes mitosis depend only on the maximum external drug concentration in which the cell has been exposed to. Note that this is an oversimplification of the complex dynamics and the adaptive response of cells occurring during and after treatment.

The cells are placed on the computational lattice with two different initial configurations. The first configuration mimics the monolayer *in vitro* experiments, with low cellularity (sparse cell distribution) and homogeneously distributed drug and the second, mimics a central plane slice of the spheroid with higher cell density and drug distribution described by a reaction-diffusion equation. The first configuration is used only to validate the correctness of the derived dose-dependent and drug-dependent probabilities (equations S1, S2) and demonstrated how the drug-response curve changes as we vary the cellularity (Figure S8). In all the simulations presented (unless stated explicitly), we use the second (dense) configuration.

At the beginning of the simulations, each cancer cell is randomly assigned an age, which corresponds to the time spent in the cell-cycle and increases at each update of the model, until the cell completes mitosis and divides. In order to avoid synchronization artefacts and account for the natural variability in the proliferative capacity of the cell population, we introduce slight heterogeneity in the cell-cycle duration. Specifically, the doubling time is randomly selected from a normal distribution with a mean of τ=22h and a standard deviation of 1h. For simplicity, the oxygen and nutrient limitation within the spheroid mass are not considered in the current formulation. Thus, mitosis occurs if there is available free space in the 3-Moore neighborhood, otherwise the cell becomes quiescent due to contact inhibition. The neighborhood reflects the maximum distance over which a cell can push other cells away in order to proliferate. The divided cells reset their age. At the time a cell is ready to divide, a probability is applied to decide whether the cell will complete mitosis, die or pause mitosis based on the drug under study and the concentration of which the cell is exposed to (see Figure S7). The proliferation rate, proliferation depth and spontaneous cell death are calibrated to fit the dynamics of the untreated spheroids. Contact-inhibited cells resume their mitosis in case free space near them becomes available.

**Computational parameters**

**Initial spheroid size**

Applying the respective estimated probabilities of drug-induced cell fate in a low cell density distribution of cancer cells, the dose-response curve (population inhibition across a variety of doses) of each drug is reproduced. Here, we explore how different initial spheroid sizes (compact configuration of cancer cells) might affect drug effectiveness. In order to keep it simple, in this set of experiments drug gradients are not considered. Note that in all other simulations, the initial radius is taken according to Table S1.

As expected, we observe that the effectiveness of a drug is considerably affected by the initial spheroid size. In Figure S8, the larger the radius of the spheroid is, the more it diverges from the 2D dose-response curve to lower inhibition levels. Smaller sized spheroids better approximate the inhibition levels of the 2D experiments. The differences observed are attributed to the increased space competition (contact inhibition) of larger spheroids, which result in relatively smaller number of proliferating cells (limited to the proliferative zone) in which the anti-mitotic drugs target.

**Lysis period**

We varied the lysis period in both a cytotoxic and a cytostatic drug with the same 2D-endpoint dose-response curve (i.e. the *in vitro* dose-response of DOX) and explored dynamically the spheroid expansion. In order to evaluate the net effect of lysis, we neglect the drug gradients within the spheroid volume, similarly to the previous set of experiments.

While lysis period plays no role in 2D cell populations (or small spheroid sizes), it plays an important role in 3D spheroids. Overall, we observed that rapid lysis enhances drug effectiveness prolonging response to therapy particularly at high drug concentrations. In contrary, for smaller doses (2D inhibition levels below 80%) increased lysis period slightly promotes drug effectiveness. Assuming a prolonged lysis period, cells still occupy space, enhancing contact inhibition. Thus, fewer cells proliferate and therefore a reduction on both the spheroid expansion and viable cell population is observed. However, this affects the efficacy of the drugs that act on proliferating cells leading to an interesting interplay that eventually enhances spheroid expansion and viability at high doses and suppresses it at lower doses. Lysis has profound effect on the effectiveness of a cytotoxic drug where dying cells prevail (Figure S9), and only slightly affects the dynamic response of spheroids exposed to a cytostatic drug (not shown). Lysis period also affects the distribution and concentration of necrotic cells, as it allows their accumulation for longer period (Figure S9-bottom).

**Drug latency period**

We applied various latency periods to DOX treated spheroids. This period depends on the cell line, the given dose, and the culture conditions and can vary from cell to cell. For simplicity, we assume that all cells in our population need the same internalization period ranging between 24 and 48 hours, and we apply this latency only in the beginning of our simulations, when cells are exposed to DOX for the first time. We also assume the same latency period to hold in 2D and 3D cultures and be independent of the drug dose. During the latency period, the cell population follows the untreated growth. For each concentration, the response probabilities are readjusted in order to achieve the same 2D endpoint inhibition level including the latency period of free, untreated growth. Increasing the latency period, the probability of cells to be affected by a given concentration of DOX increases as well, as the same inhibition level should be reached in less time. In these experiments, we keep the initial spheroid conformation fixed and the diffusion gradients of the drug the same.

As can be seen in Figure S10, increasing the latency period from zero (dotted lines) to 48 hours (solid line), a dramatic increase in the temporal response to DOX is observed. For 2D inhibition levels above 90%, contrary to the scenario where the latency period is zero, no spheroid regrowth is observed when the latency period equals to 48h.

**Drug penetration**

Drug gradients can be formed by variations in the drug diffusion coefficient and the drug uptake rate. Thus, in general, the drug penetration length depends on the cancer cell type and the experimental settings. In particular, the drug diffusion may depend on spheroid-generation techniques and culture medium as can affect several factors including the medium of the intercellular space between cells and the compactness of tumor spheroid. As expected, the simulations show that the formation of drug diffusion gradients within the spheroid volume affect the drug effectiveness. Drugs with higher penetration lengths prolong their response to therapy (Figure S11). As already mentioned, in our simplified modelling setup, in which the drug-induced cell fate depends on the external drug concentration, imposing drug gradients in the system directly alters the drug-induced effect on the cell. Thus, the drug gradients can be used as a coarse control parameter that alters the sensitivity of the cells to treatment.

**Cytostatic/cytotoxic assumption**

We also investigated whether differences can be observed in the temporal dynamics of treatment between a drug that acts as cytostatic and a drug that acts as cytotoxic, given the same endpoint dose-response curve. By construction, both drug behaviors are modelled to achieve the same inhibition level of the viable cell population at the end of the exposure time (72h). Yet, for the same inhibition level, the probability of a cell to pause mitosis is different from the probability to die because cells in the cell cycle-arrest state accumulate over time counting on the viable population.

We varied the probability λ from zero (cytotoxic) to one (cytostatic) in order to explore how these cell states affect the spheroid expansion. The probability λ is applied on the same 2D-endpoint dose-response curve (i.e. the *in vitro* dose-response of DOX). We can observe that at higher concentrations, the cytostatic drug is more effective relative to the cytotoxic drug, reducing the spheroid expansion and prolonging the response period (Figure S12). Note, however, that if we apply the same probabilities for a cell to pause mitosis and for a cell to die, then the cytotoxic drug is superior relative to the cytostatic drug at high probabilities.

**Drug dilution rate**

The simulations follow the *in vitro* protocol where the treatment period is set to 72h and 50% medium replenishment is performed (the drug concentration is reduced by half) every two days post-treatment (see also schematic overview in Figure S1D). As shown in Figure S13 for the DOX example, lower elimination rates of the drug from the medium increase the spheroid responsiveness to the treatment.

**Drug dominance in combination (intersection probability)**

If drug A is cytotoxic and drug B is cytostatic, the cell fate (i.e. cell death or cell cycle-arrest) at the intersection ($P\left( A\cap B \right)$) of the probabilities is unknown. Considering that cell death and cell cycle-arrest affect differently the spheroid dynamics, it is important to explore whether these differences have any profound effect on the drug concentrations in combination. As can be seen in Figure S14**,** only slightly differences are observed. JPS stands for the joint probability split and if equals to zero, cell death occurs at the intersections. JPS equal to 1 corresponds to cell cycle-arrest and when JPS is equal to 0.5 there is equal probability of a cell to undergo cell-cycle arrest and die at the intersection.

**Compare *in vitro* combination experiments with null models**

In principle, synergy is analysed by comparing the experimentally observed effect of the combined drugs under study with the mathematical reference effect of a null model. The null models are derived based on the effects of the individual drugs under specific assumptions. If the experimental effect significantly exceeds the effect predicted by the null model, synergy is detected.

The aim of our work is to develop a spatiotemporal model for predicting drug responses in monotherapy and in combination therapy (under the null hypothesis of drug independence) providing a mechanistic description of the main bioprocesses involved at the level of cells. Our proposed null model of drug combination has been calibrated to fit the monotherapy treatment response of the *in vitro* spheroids.

In Figure S15, the *in vitro* combination experiments (blue bars) are compared to the response additivity (red bars), the Bliss independence model (yellow bars) and our mechanistic null model (purple bars) at various dose pairs and at different points in time. The respective mean values of the inhibition levels for each case are depicted in Table S2.

**Compare *in vitro* monotherapy and combination experiments varying the cytotoxic-cytostatic assumption for DOX and TMZ**

For simplicity, we have described DOX as purely cytotoxic and TMZ as purely cytostatic in order for the related probabilities to be estimated. However, we acknowledge that it is expected that neither of the two drugs is purely cytostatic or cytotoxic, and that this effect is likely cell line-dependent, dose-dependent, condition-dependent (hypoxia, etc), as well as time-dependent.

We simulated a scenario where TMZ is also purely cytotoxic (Figure S16). Furthermore, we computationally explored ranges between these two extreme scenarios of pure cytotoxicity and pure growth inhibition for either of the two drugs. In Figure S17, we show a scenario where DOX is predominantly cytotoxic (λ=0.4) and TMZ is predominantly cytostatic (λ=0.6). The model has been recalibrated to describe 3D monotherapy for this range of values. Changes in these assumptions did not alter the main conclusion regarding the *in vitro-in silico* discrepancy of combination treatment.

**
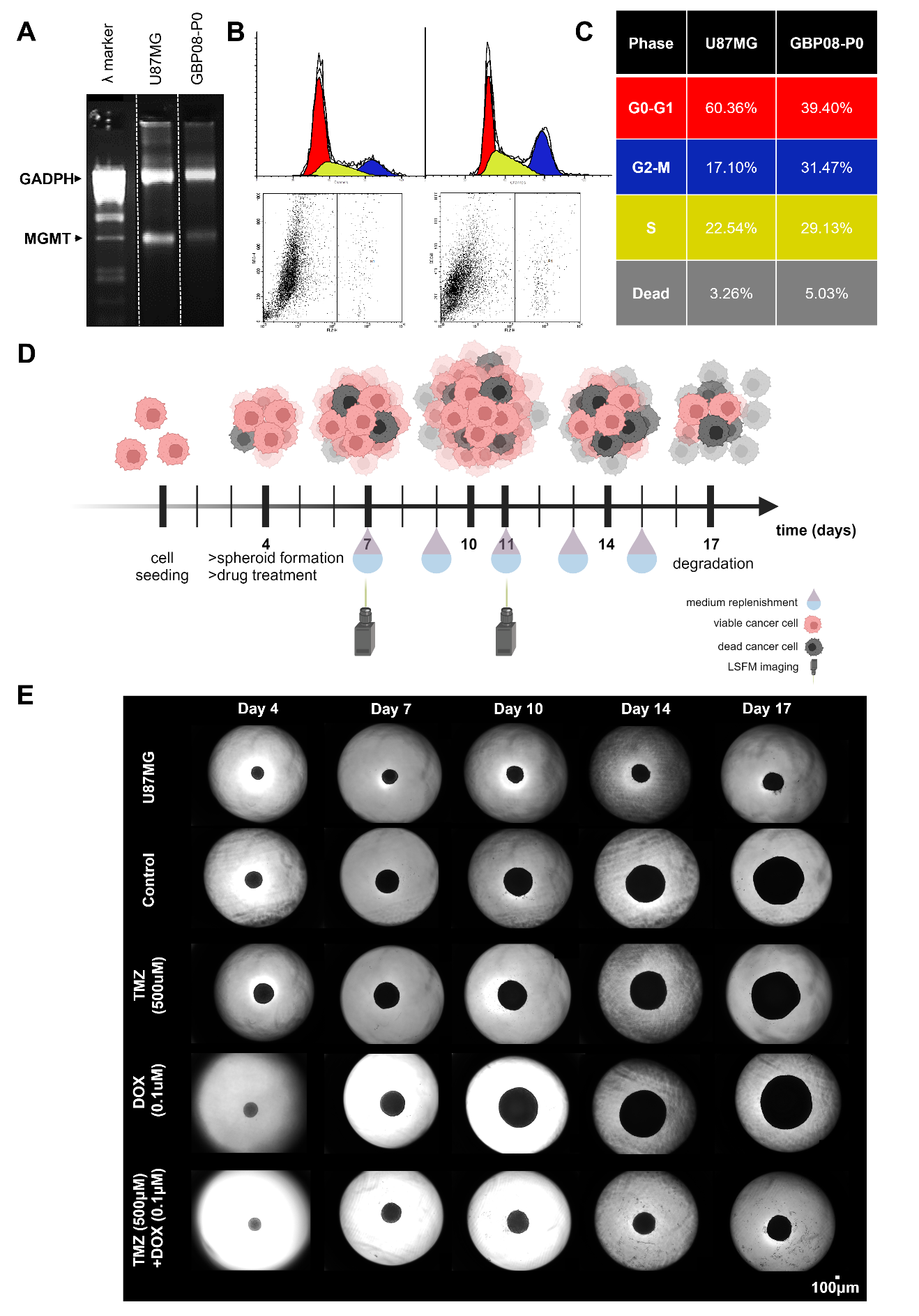
**

**Figure S1. A.** Northern blot of extracted RNA (left) and RTpcr mRNA quantification (right) of the GBP08-P0 primary cell line for the MGMT promoter status, as opposed to the U87MG secondary cell line. The different samples are denoted with dashed lines. The original gel is presented in the appendix.

**B.** Cell cycle distribution results for the U87MG cells (left column) and the GBP08-P0 primary cells (right column) for the cell cycle phases (upper row) and the dead cells (low row), respectively. Indicative percentages are denoted on the table **C**. Note that the estimation of death and the cell cycle phases originate from different experiments. **D.** Schematic overview of the experimental design, depicting the main procedures that generated spheroids undergo during treatment. **E.** Brightfield images of representative spheroids at different timepoints for the different treatment conditions. U87MG spheroids are used as a growth reference. In the final timepoints of the combination treatment scheme notice the debris of dead cells around the spheroids. Images were captured at 4x magnification. Scale bar is set at 100 microns.

**Figure S2. Drug treatment of the U87MG cell line.** Brightfield images of representative spheroids at different timepoints for the different treatment conditions. Notice that U87MG spheroids are more resistant to DOX and although debris of dead cells around the spheroids can be seen right after treatment (Day 4), only after day 15-17 the spheroid is
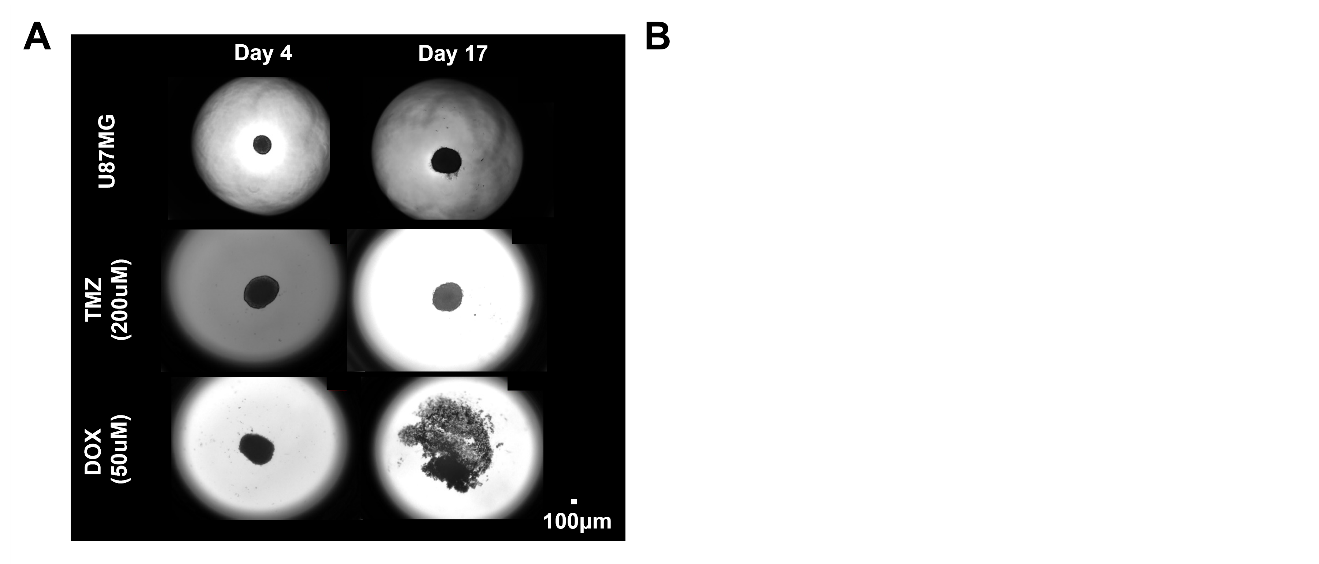
 completely non-viable. For most Temozolomide concentrations tested, no difference to control spheroid growth was observed. Images were captured at 4x magnification. Scale bar is set at 100 microns.


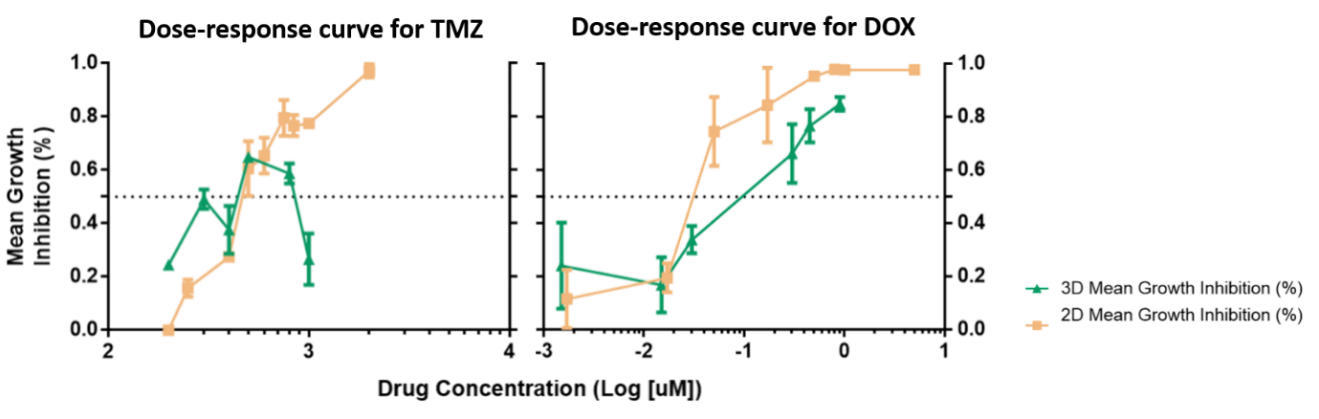


**Figure S3. Dose response curves for TMZ- and DOX-treated GBP08-P0 primary** GB **cells in 2D and 3D cultures.** Notice that there is no sigmoidal pattern for the TMZ-treated spheroids curve; GB cells are more sensitive in TMZ treatment as monolayers.

**
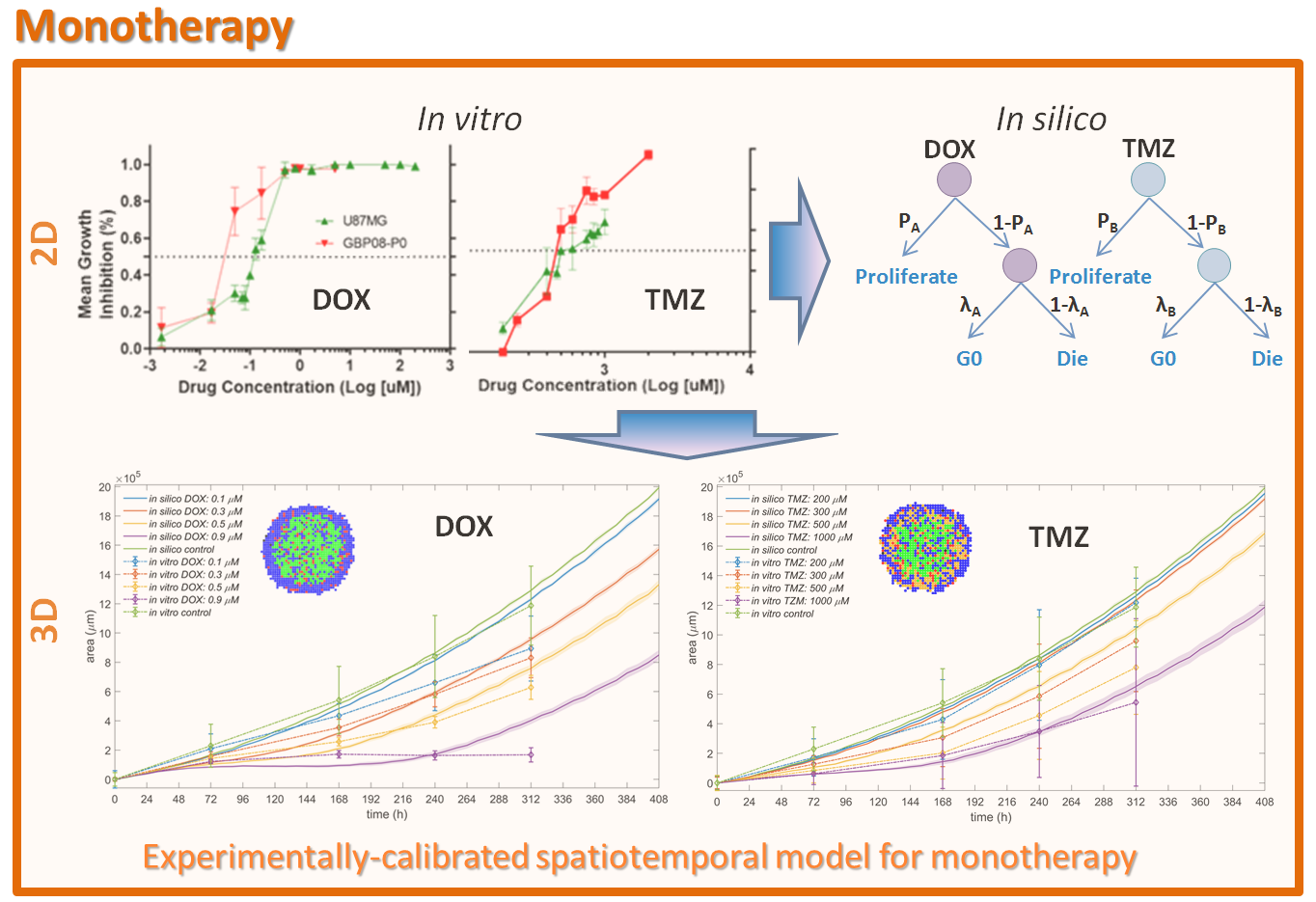
**

**Figure S4. Illustrative framework – monotherapy.** (First row) The dose-response curve of each drug is converted to a dose-dependent probability for an event (i.e. cell death or cell-cycle arrest) to occur. (Second row) The temporal evolution of the untreated and treated *in vitro* spheroids at various doses of each drug along with the estimated dose-dependent probabilities are used to calibrate the monotherapy 3D models accounting also for drug diffusion and space competition among cells.

**
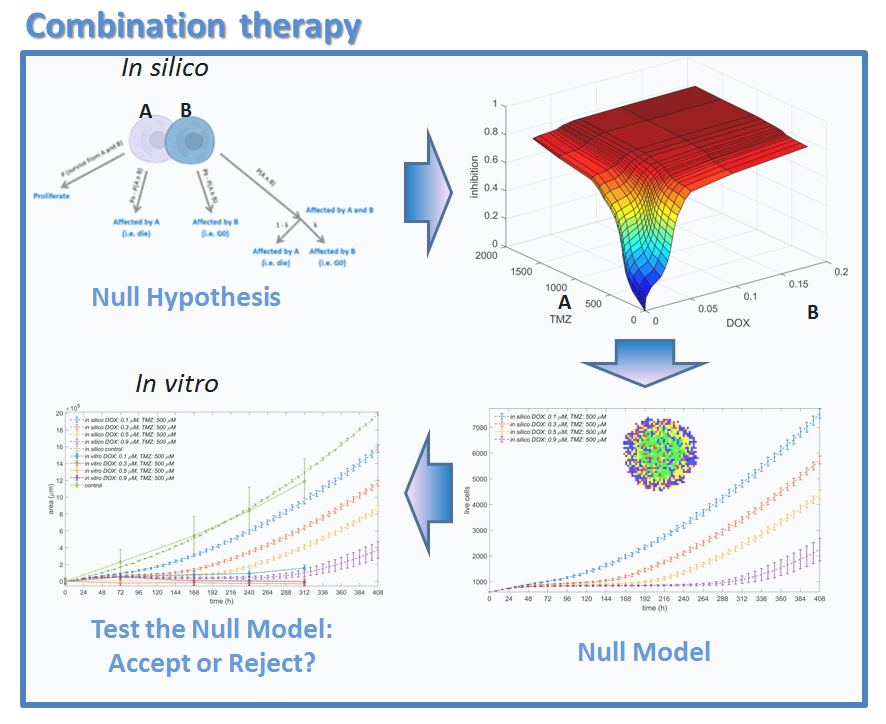
**

**Figure S5. Illustrative framework – combination therapy.**

(First row) The combination probability under the drug independence assumption is mathematically formulated from the probabilities derived in monotherapy. Various concentrations of drug A can be tested against various concentrations of drug B and their estimated combined inhibition is shown. (Second row) All the parameters used to describe the 3D monotherapy are used to describe the 3D combination therapy yet accounting now for the combination probabilities. The 3D null model is then compared to the 3D *in vitro* experiments. Alternative descriptions are tested based on the parameter study in an iterative manner aiming to fit both monotherapy and combination therapy experiments before a rejection or acceptance of the hypothesis.

**
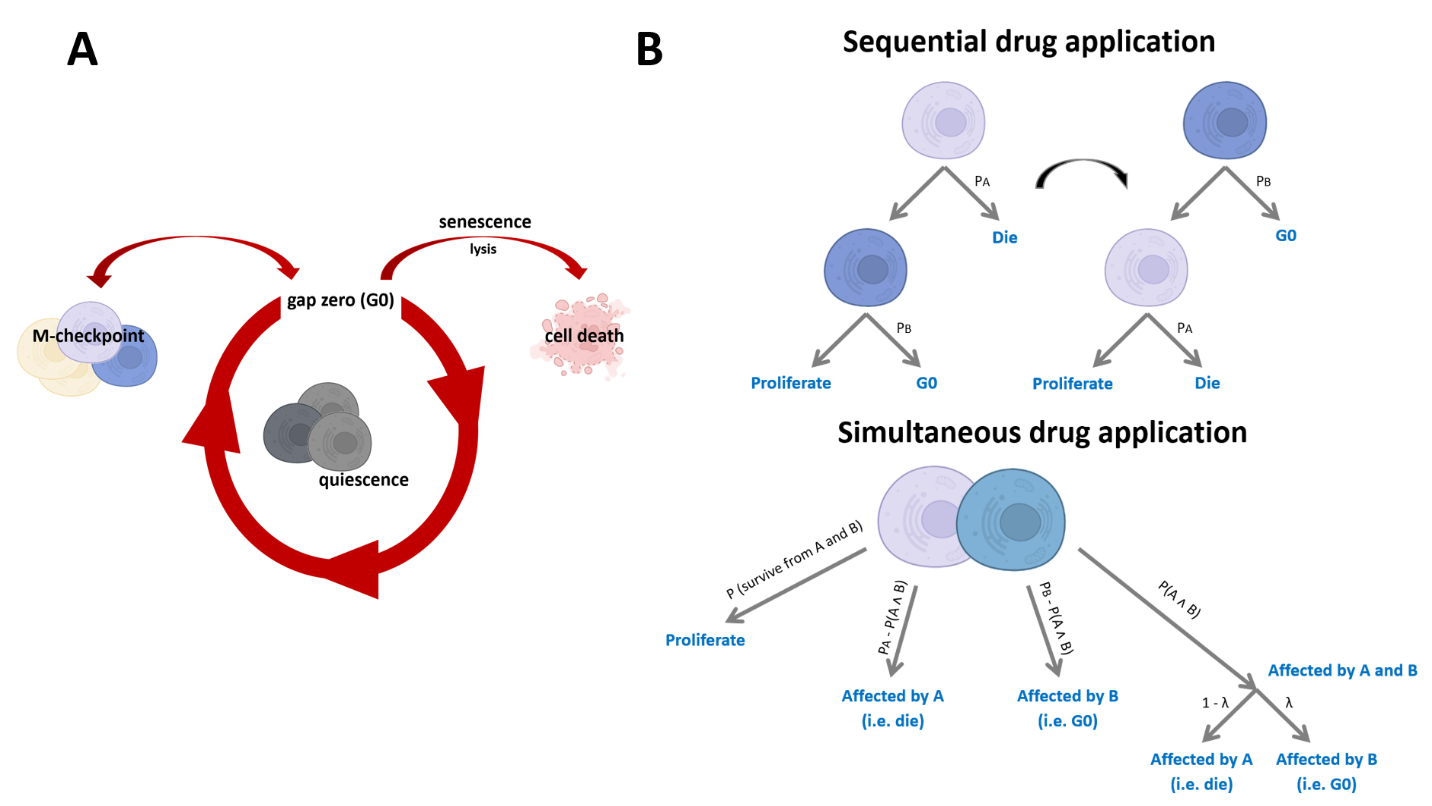
**

**Figure S6. Cell fate assumptions.**

A. Illustrated representation of cancer cells that undergo G0 phase. Non-dividing cells will become quiescent or senescent (a.k.a. irreversible quiescence) and eventually die according to the time spent in G0.

B. Probabilistic cell fate according to the drug presence in monotherapy or in combination. Drug A is assumed as cytotoxic and Drug B as cytostatic, respectively. In general, two drug applications can be considered in drug combination, sequential or simultaneous, respectively.


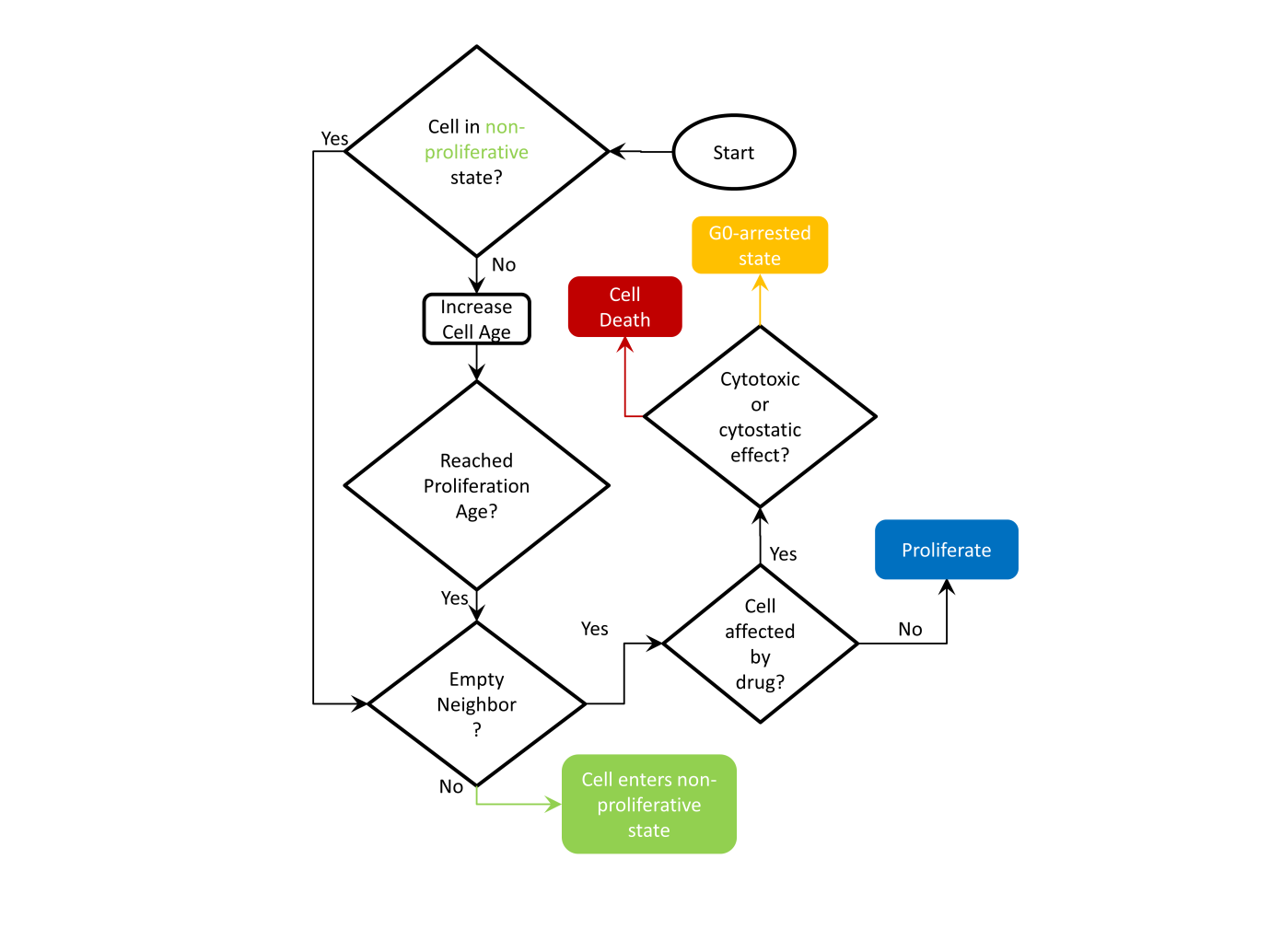


**Figure S7. Discrete cell flow chart.** Cells can proliferate (blue box), become quiescent due to contact inhibition (green box), and experience drug-induced senescence (yellow box) and die (red box).


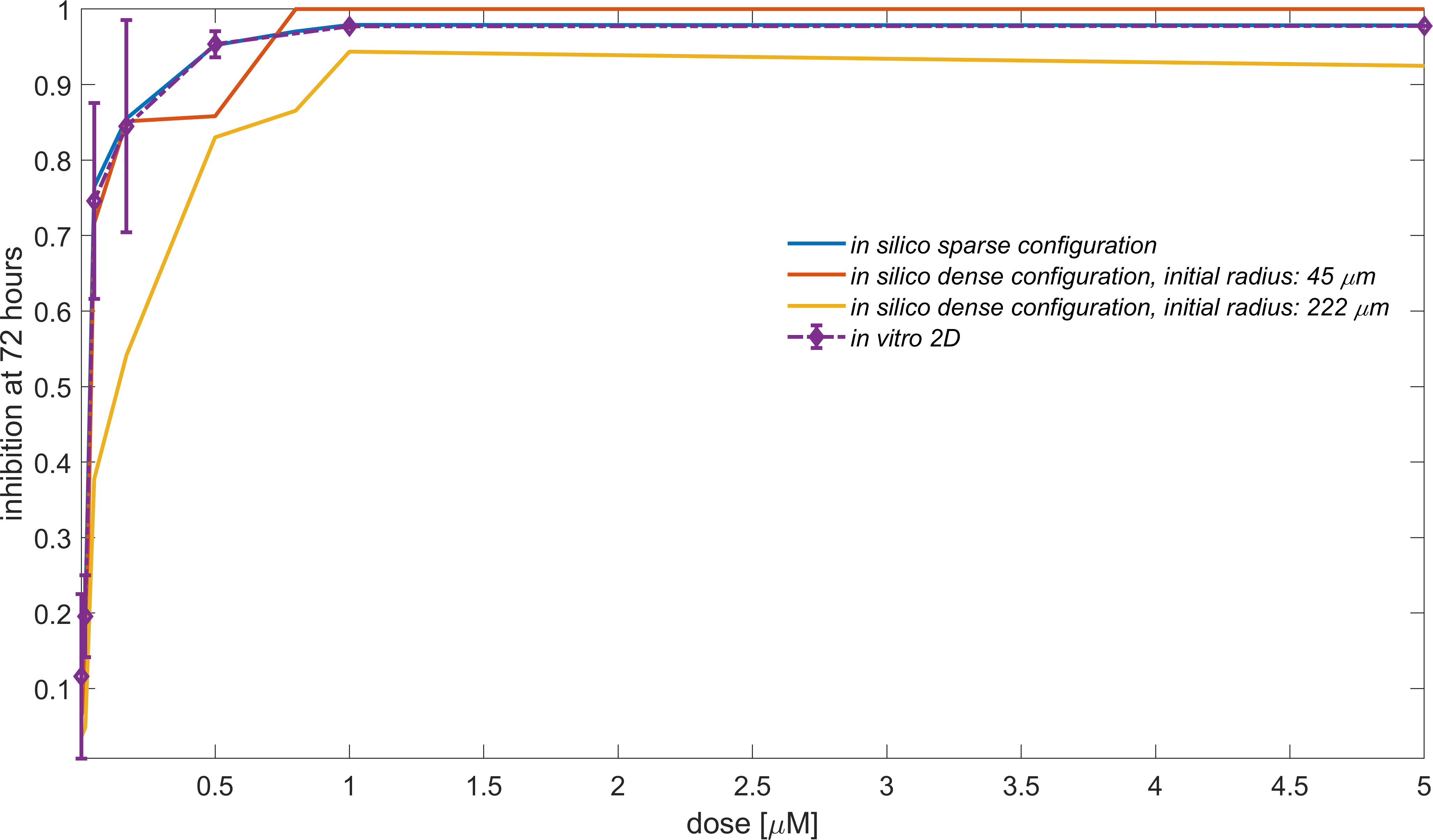


**Figure S8. Effect of initial spheroid size in dose-response curves.** Applying the derived probabilities in a cancer population in sparse configuration the mean dose-response curve (population inhibition across a variety of doses) of DOX (purple line) is reproduced *in silico* (blue line). Compact circular configuration of cancer cells mimicking a central slice of a tumor spheroid affects drug effectiveness. Red and yellow lines correspond to initial radius of 45 μm and 222 μm, respectively.


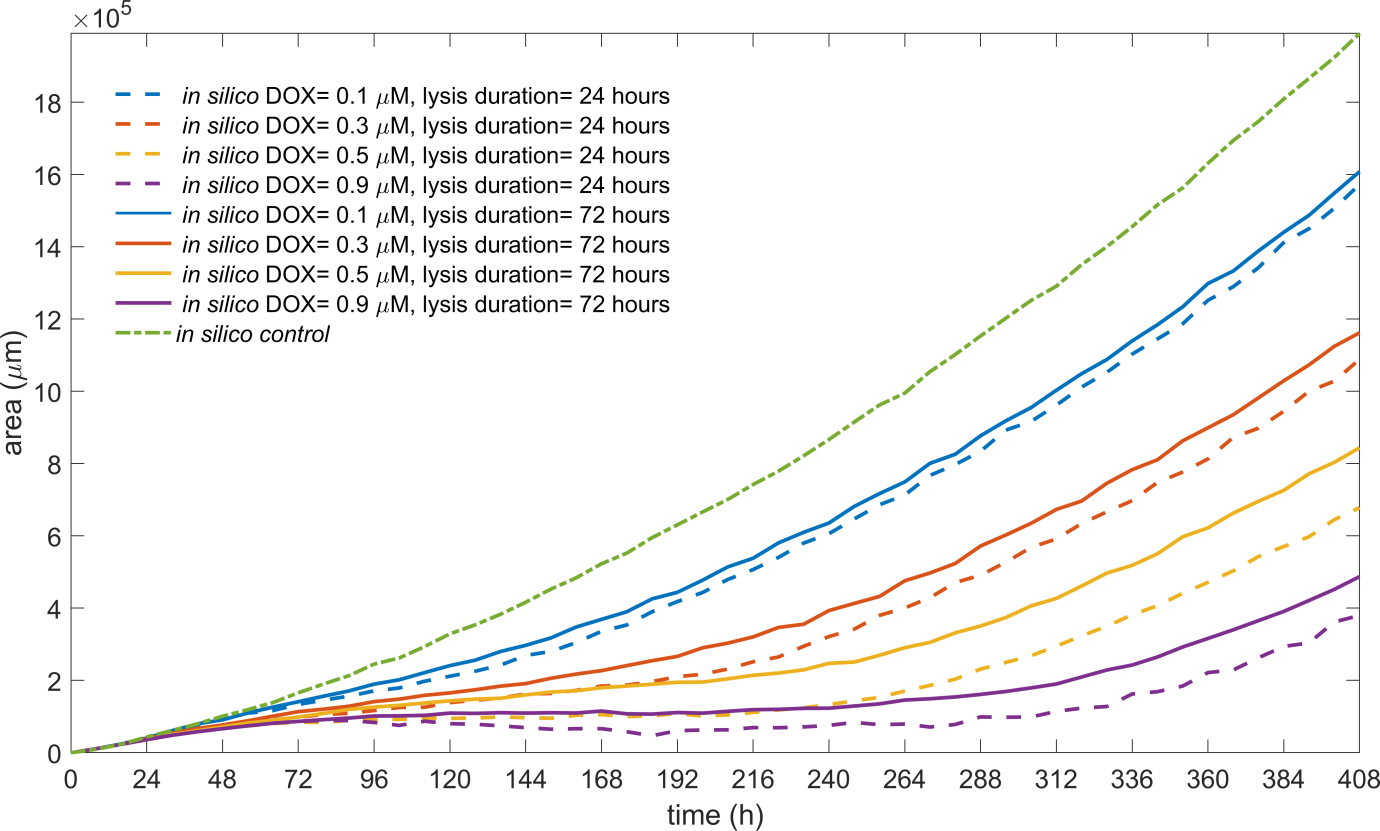


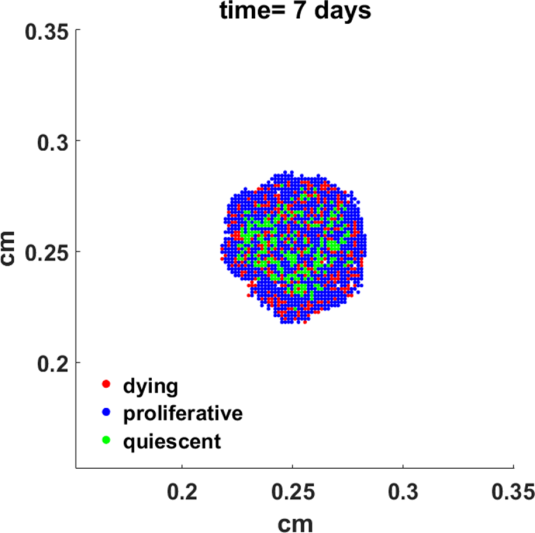

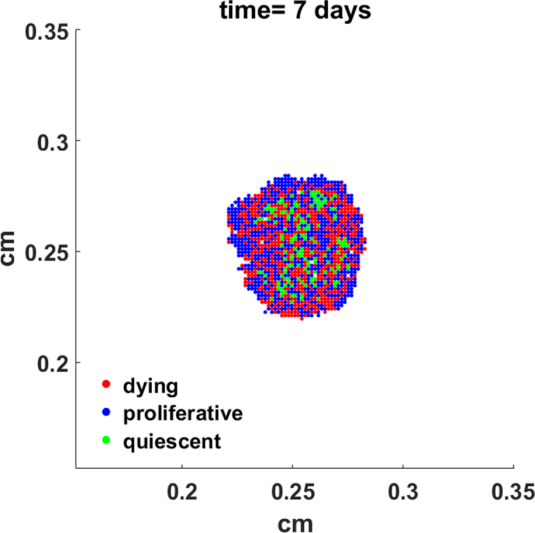


**Figure S9. The predicted effect of lysis period on the temporal evolution of spheroid expansion, under the exposure of cancer cells in a cytotoxic drug.** Rapid lysis enhances drug effectiveness of a cytotoxic drug at high doses. The predicted effect of lysis period on the spatial distribution of cells, under the exposure of cancer cells in a cytotoxic drug. DOX concentration equals to 0.2 μM. The snapshots are taken at day 7: (left) lysis period equals to 24h, (right) lysis period equals to 72h.


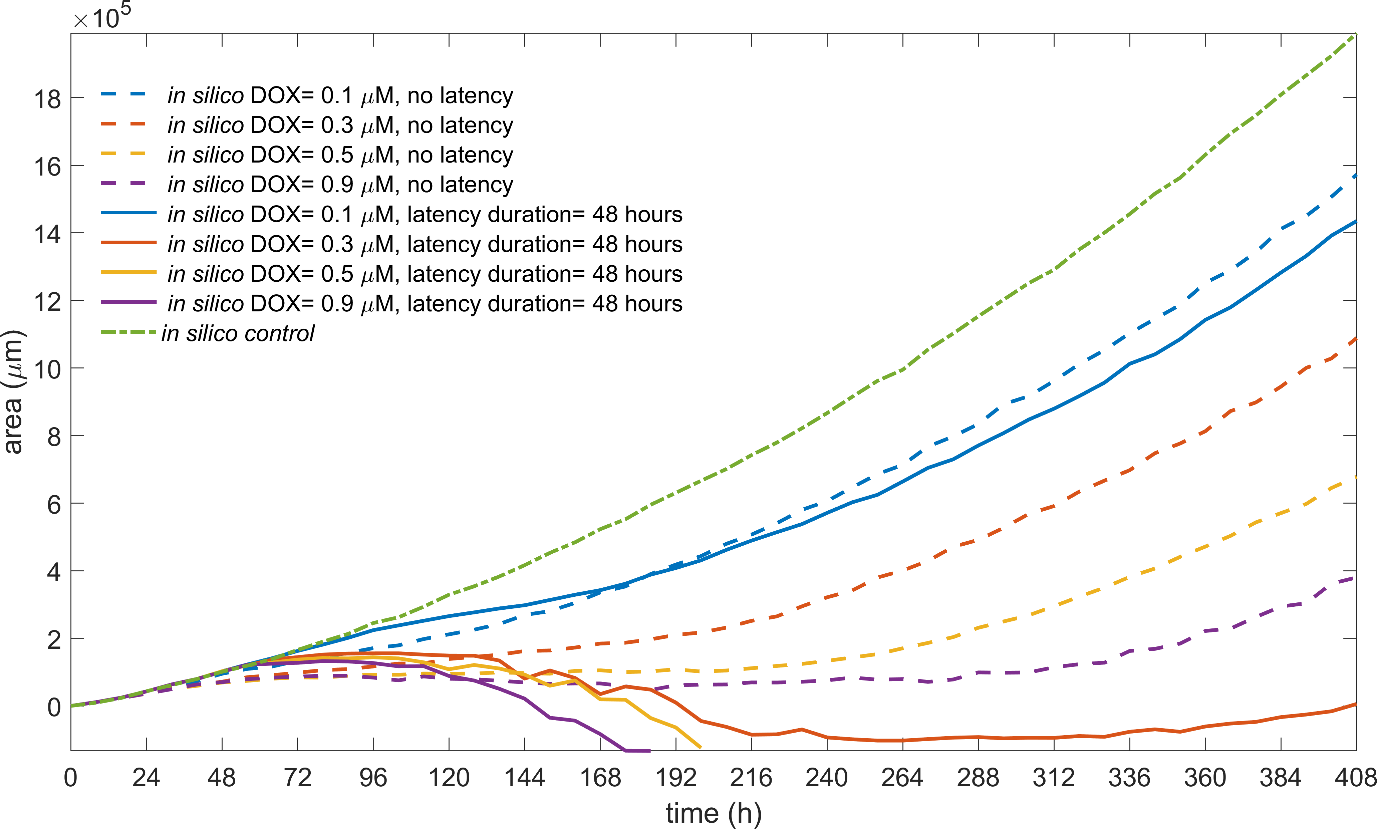


**Figure S10. The effect of latency on the temporal evolution of spheroid expansion response for various concentrations of DOX.** Latency period is zero (dotted lines) and latency period is 48h (solid lines).


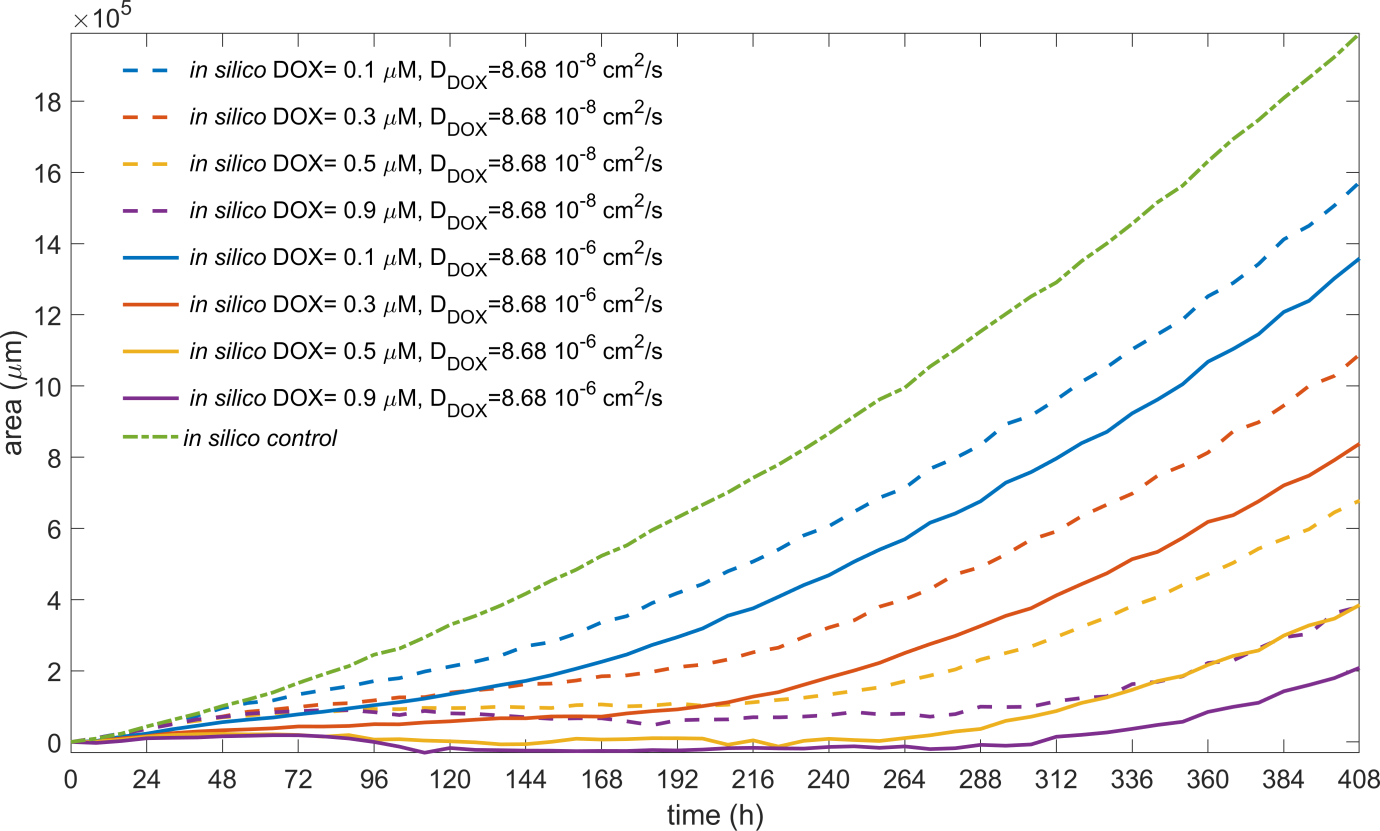


**Figure S11. The effect of drug diffusion on the temporal evolution of spheroid expansion response for a given uptake rate under various drug concentrations.** Low diffusion coefficient (dotted lines), and high diffusion coefficient (two orders of magnitude difference) depicted with solid lines are illustrated.


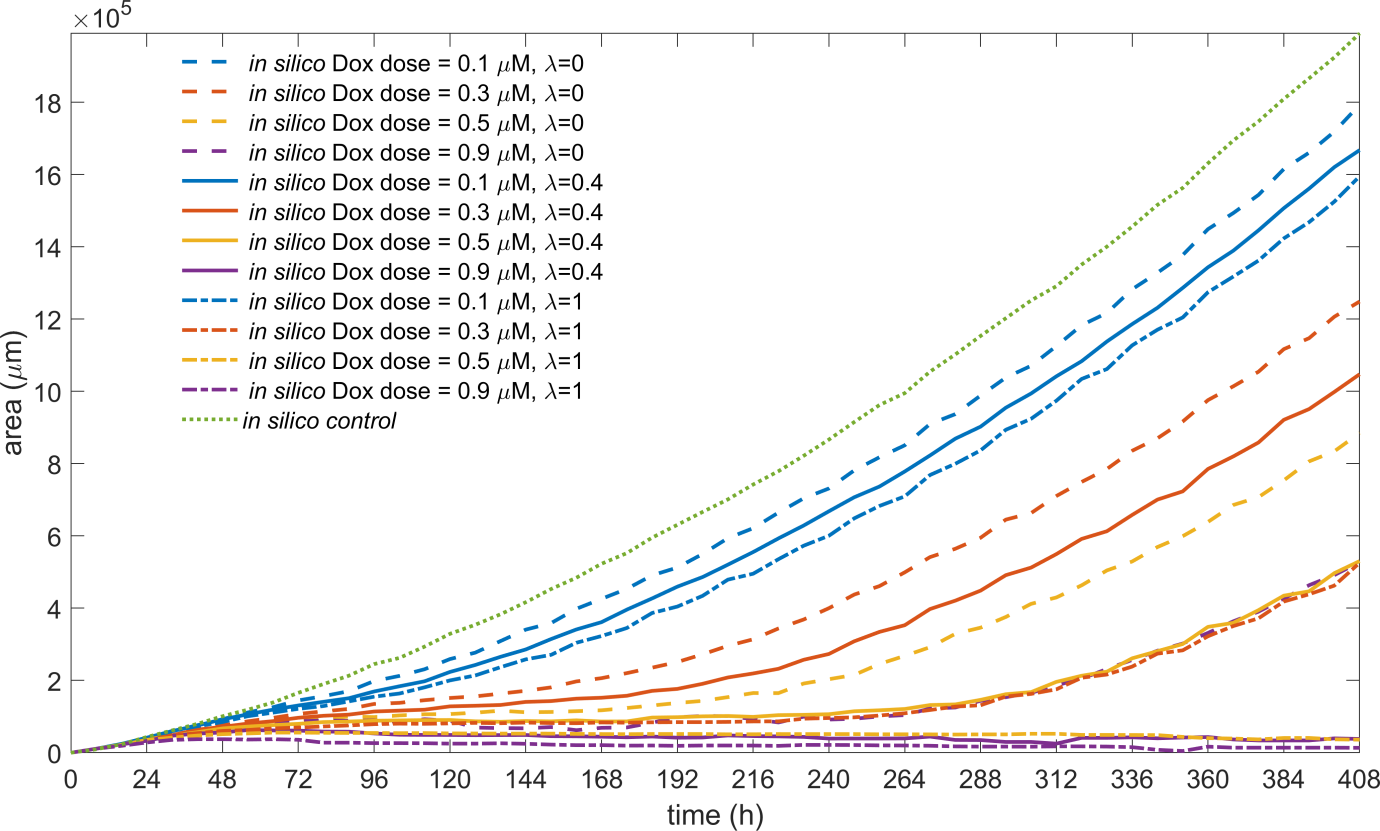


**Figure S12. The effect of the cytotoxic versus the cytostatic drug mechanism on the spheroid expansion response.** We show three cases of the same drug assumed to act as solely cytostatic (λ=1), solely cytotoxic (λ =0) and both 40% cytotoxic/ 60% cytostatic (λ=0.4).


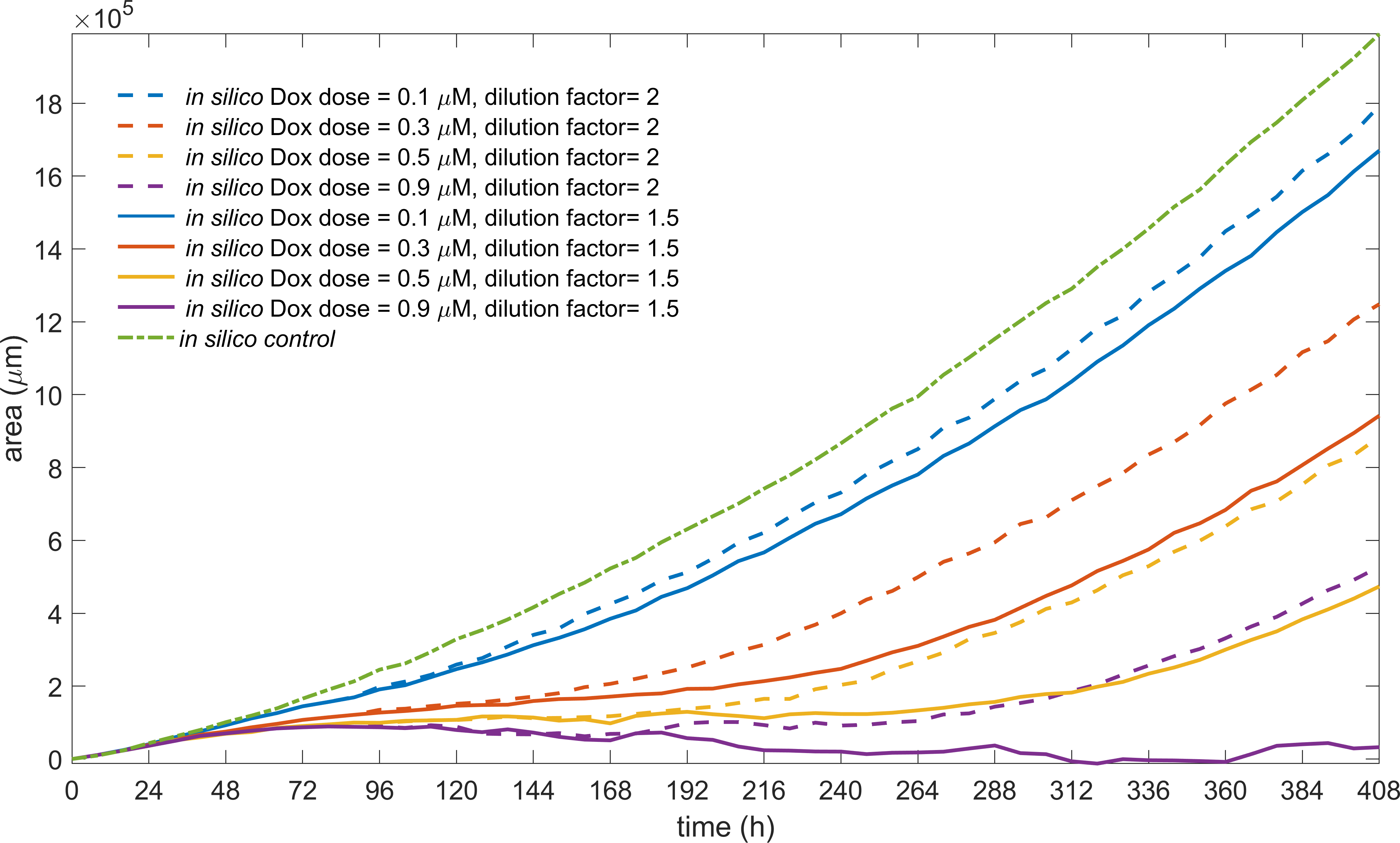


**Figure S13. The effect of drug dilution for DOX monotherapy treatment.** Two cases are illustrated; the drug concentration a) is 2/3 diluted every 2 days (solid lines) and b) is diluted by 2 (dotted lines).


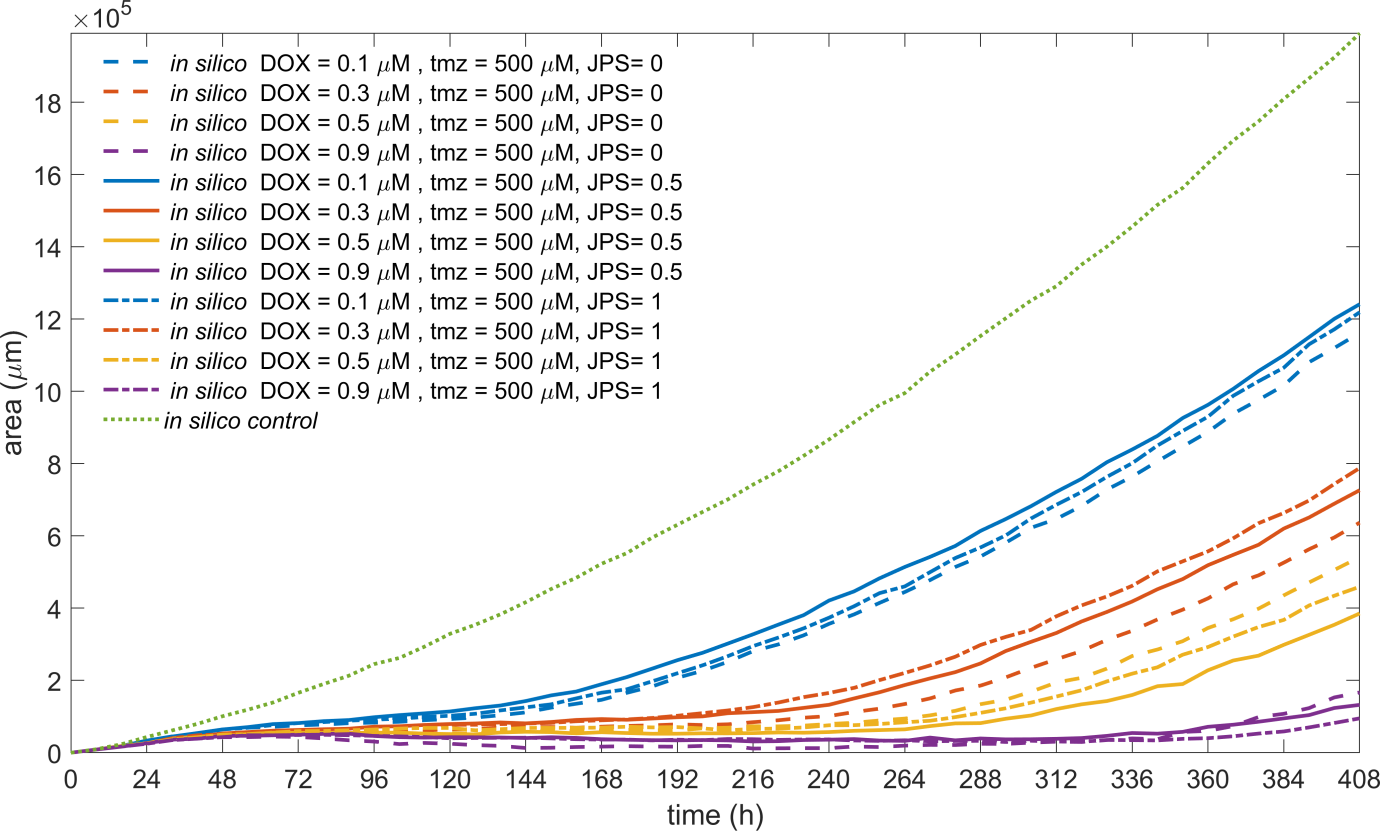


**Figure S14. Drug dominance in combination.** JPS equal to 1 corresponds to dominance of TMZ, whereas JPS equal to 0 corresponds to dominance of DOX.


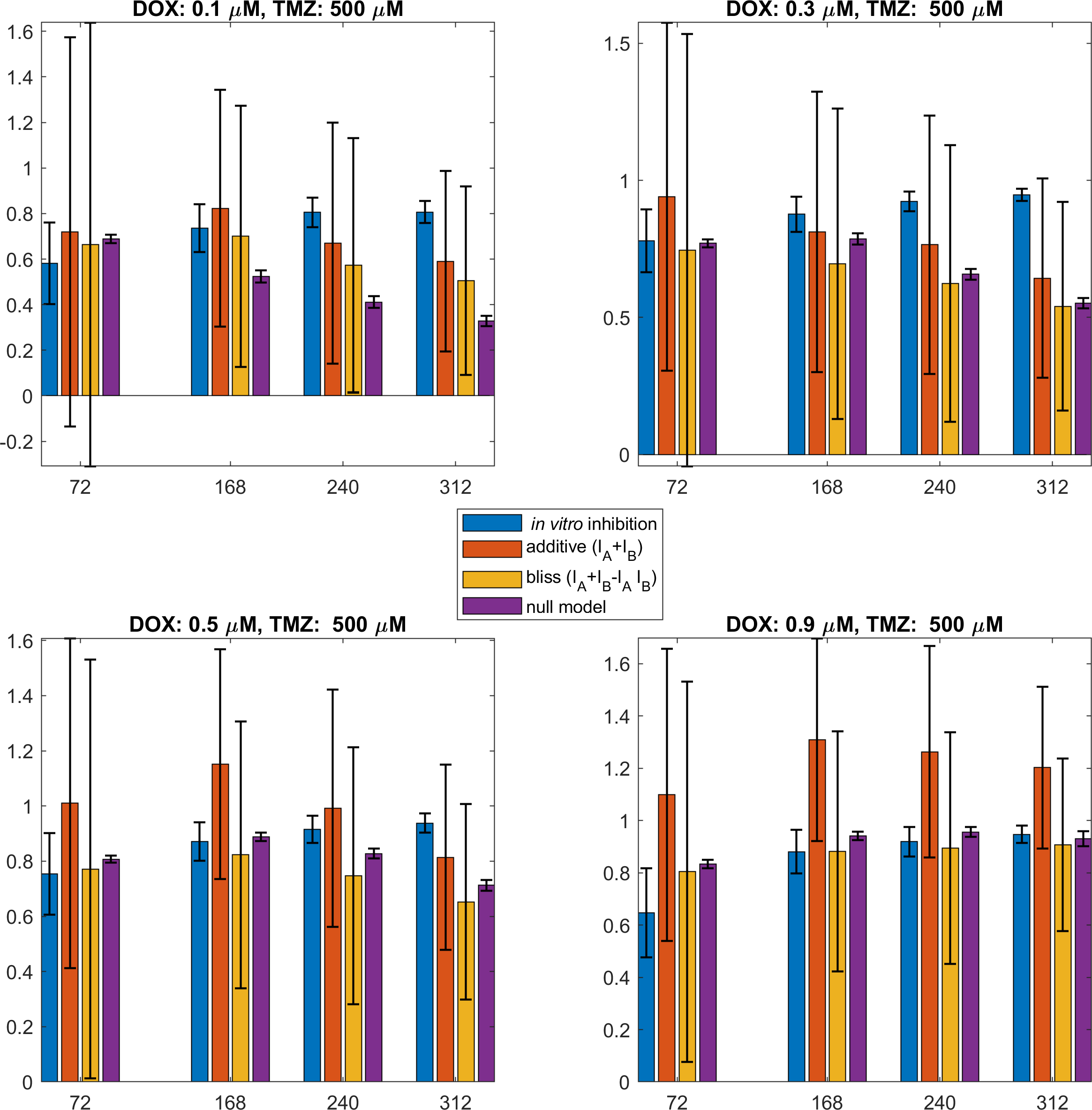


**Figure S15. Compare *in vitro* combination experiments (blue bars) with the null models:** response additivity (red bars), Bliss independence (yellow bars), and the proposed mechanistic null model (purple bars) at various drug pair concentrations and different timepoints.

**
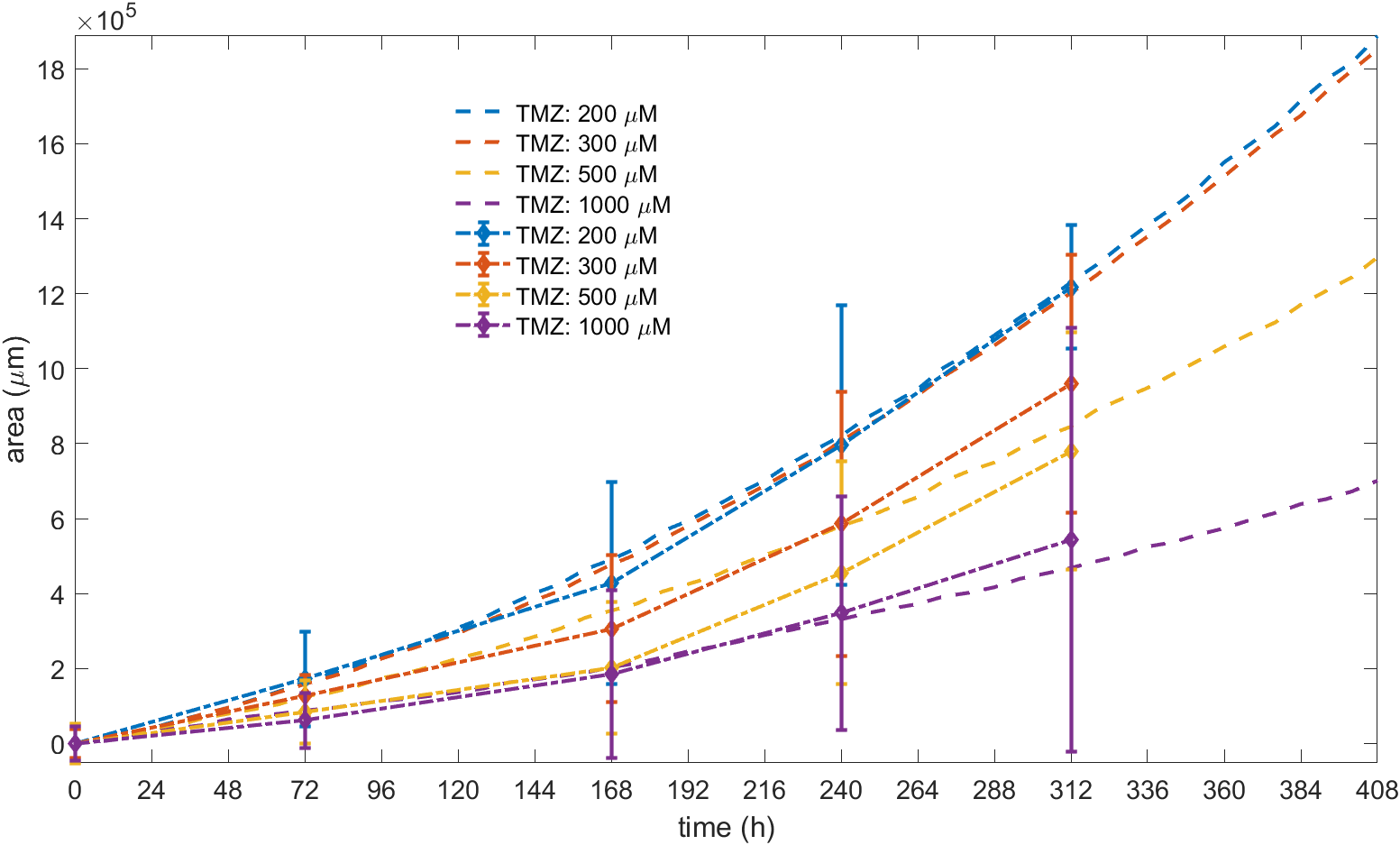
**

**
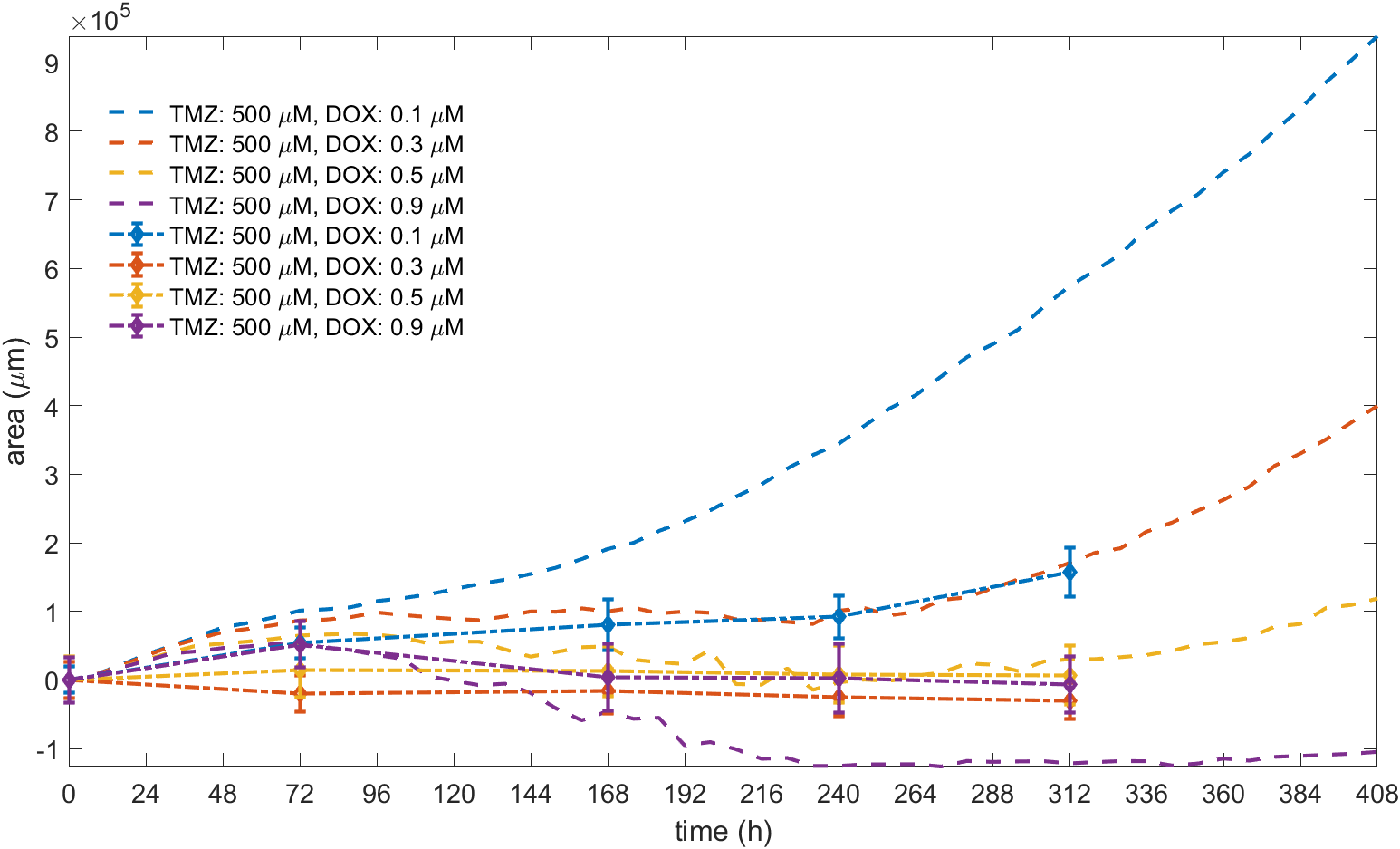
**

**Figure S16. Monotherapy and combination under the assumption that both DOX and TMZ are purely cytotoxic drugs.** A discrepancy is observed regarding the *in vitro-in silico* results in combination theory for the (DOX,TMZ) pair equal to (0.1, 500) μΜ. Note however that strong post-treatment affects have been assumed for TMZ (no dilution) to fit the monotherapy conditions.

**
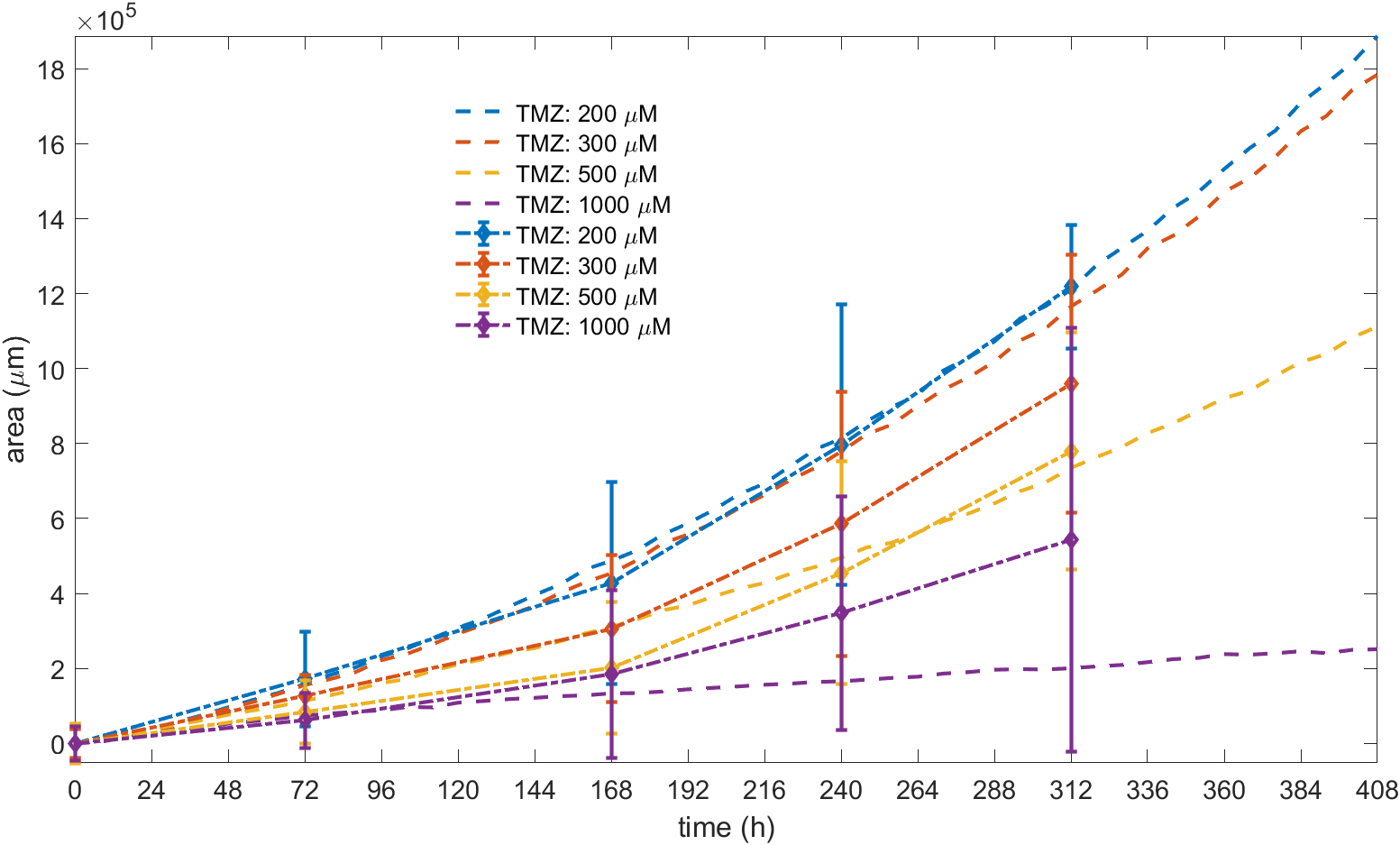
**

**
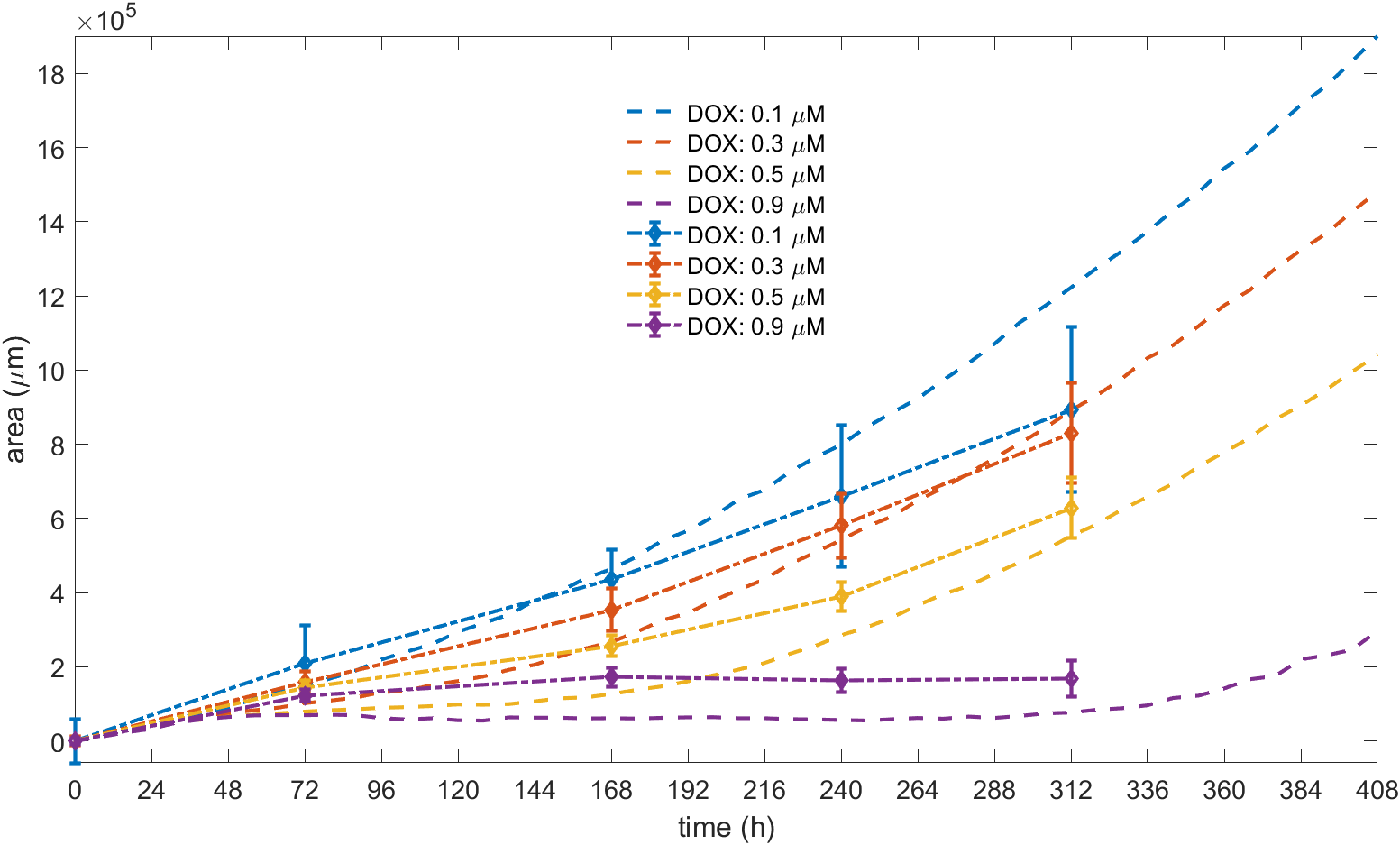
**

**
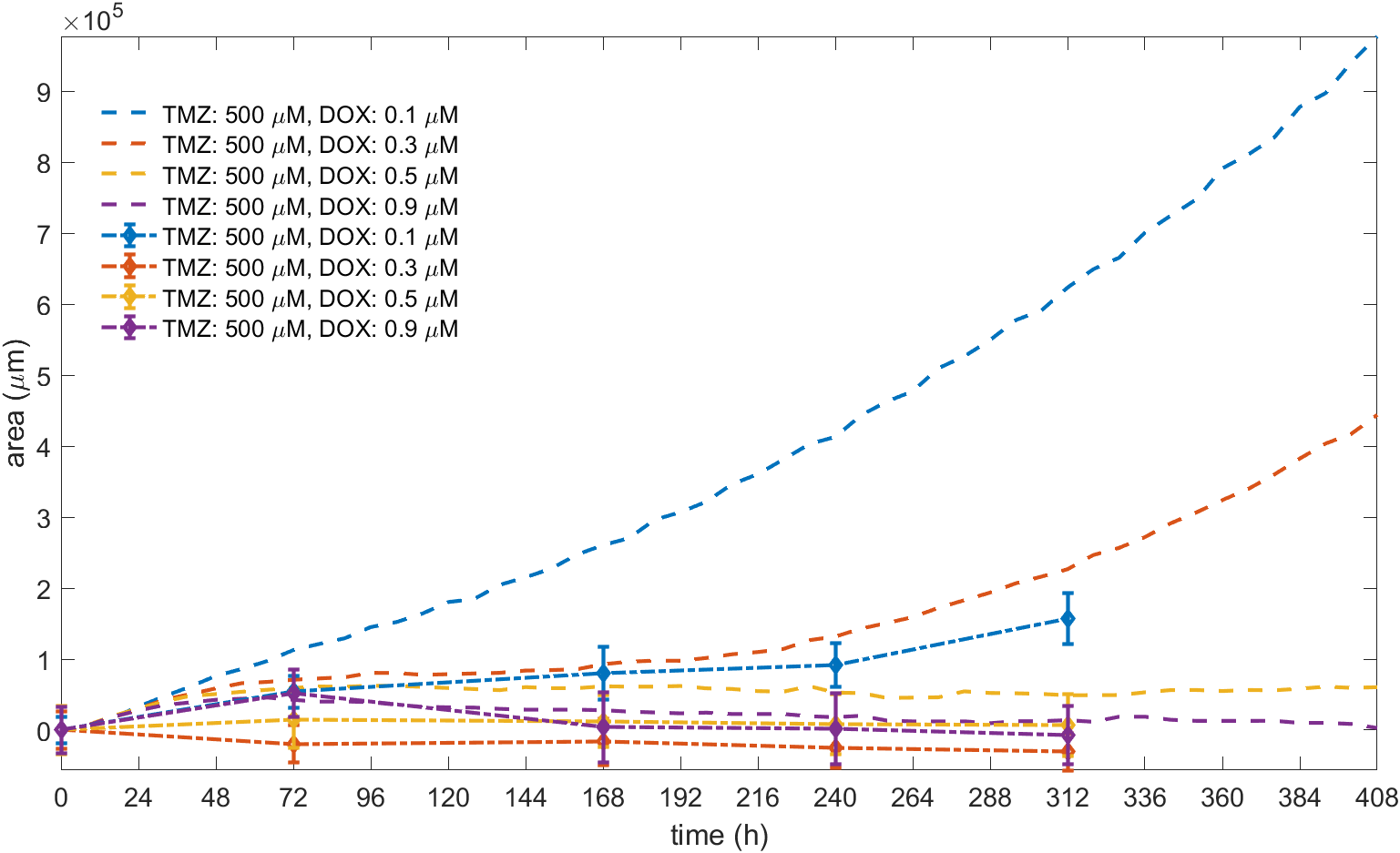
**

**Figure S17. Monotherapy and combination under the assumption that DOX is predominately cytotoxic (40% static, 60% toxic) and TMZ is predominately cytostatic (60% static, 40% toxic).** A discrepancy is observed regarding the *in vitro-in silico* results in combination theory for the (DOX,TMZ) pair equal to (0.1, 500)μΜ and (0.3, 500)μΜ. Note however that strong post-treatment effects have been assumed for TMZ (no dilution) to fit the monotherapy conditions.

**Table S1. Computational parameters assumed for the initialization of the computational experiments.**

| **Parameter** | **Value** | **Reference** |
| --- | --- | --- |
| **Cell size** | 15 μm | experimental, (4) |
| **Proliferation age** | 22h | experimental, (4) |
| **Proliferation depth** | 3 cells (= 45 μm) | experimental, (4) |
| **Random death rate** | 10% of proliferation rate | experimental, (4) |
| **Lysis time** | 24h | explored |
| **Initial size: spheroid radius** | 13.8 cells or 207 μm | experimental |
| **Initial population density** | 90% | (4) |
| **TMZ uptake** | 1.4^e-12^ μmol/(cell s) | explored |
| **TMZ diffusion factor** | 8.68^e-07^ cm^2^/s | explored |
| **DOX uptake** | 1.4^e-15^ μmol/(cell s) | explored  (based on (5)) |
| **DOX diffusion factor** | 8.68^e-08^ cm^2^/s | explored  (based on (5)) |

**Table S2. Mean inhibition in DOX-TMZ combination treatment of spheroids.** Estimations based on the *in vitro* experiments (first column) and compared with different null models at various drug concentrations and different timepoints.

| Doses | time (h) | *in vitro* inhibition | Response additivity | Bliss independence | *in silico* null model |
| --- | --- | --- | --- | --- | --- |
| DOX: 0.1 & TMZ: 500 | 72 | 0.762955006 | 0.719524777 | 0.664876233 | 0.68880324 |
|  | 168 | 0.890524711 | 0.823224357 | 0.700104031 | 0.52412367 |
|  | 240 | 0.905744911 | 0.670239232 | 0.572862766 | 0.41153357 |
|  | 312 | 0.867434033 | 0.590333434 | 0.505520558 | 0.32815297 |
| DOX: 0.3 & TMZ: 500 | 72 | 1.054065505 | 0.940889507 | 0.746067835 | 0.77031810 |
|  | 168 | 1.03069307 | 0.812157992 | 0.695974025 | 0.78653981 |
|  | 240 | 1.030234952 | 0.765242302 | 0.624420682 | 0.65741089 |
|  | 312 | 1.025571088 | 0.643541696 | 0.540465907 | 0.55287118 |
| DOX: 0.5 & TMZ: 500 | 72 | 0.935542929 | 1.010146524 | 0.771469751 | 0.80696069 |
|  | 168 | 0.976616418 | 1.151887503 | 0.822762292 | 0.88810516 |
|  | 240 | 0.990149056 | 0.991891393 | 0.747422545 | 0.82765281 |
|  | 312 | 0.994095303 | 0.813735657 | 0.652243423 | 0.71229032 |
| DOX: 0.9 & TMZ: 500 | 72 | 0.773352025 | 1.098347082 | 0.803819731 | 0.83265518 |
|  | 168 | 0.992522111 | 1.308927577 | 0.881370209 | 0.94111537 |
|  | 240 | 0.99712913 | 1.262789283 | 0.894438115 | 0.95620353 |
|  | 312 | 1.00576837 | 1.202206596 | 0.90737772 | 0.92938317 |

**SI References**

1. Stéphanou, A, Ballesta, A. pH as a potential therapeutic target to improve temozolomide antitumor efficacy : A mechanistic modeling study. Pharmacol Res Perspect. 2019;e00454.

2. Baek N, Seo OW, Kim M, Hulme J, An SS. Monitoring the effects of doxorubicin on 3D-spheroid tumor cells in real-time. Onco Targets Ther. 2016 Nov 22;9:7207-7218. doi: 10.2147/OTT.S112566. PMID: 27920558; PMCID: PMC5125797.

3. McKenna, M.T., Weis, J.A., Barnes, S.L. et al. A Predictive Mathematical Modeling Approach for the Study of Doxorubicin Treatment in Triple Negative Breast Cancer. Sci Rep 7, 5725 (2017).

4. M. E. Oraiopoulou *et al.*, In Vitro/In Silico Study on the Role of Doubling Time Heterogeneity among Primary Glioblastoma Cell Lines. *Biomed Res Int* **2017**, 8569328 (2017).

5. G. Tzedakis, E. Liapis, E. Tzamali, G. Zacharakis, V. Sakkalis, A hybrid discrete-continuous model of in vitro spheroid tumor growth and drug response. *Conference proceedings : ... Annual International Conference of the IEEE Engineering in Medicine and Biology Society. IEEE Engineering in Medicine and Biology Society. Conference* **2016**, 6142-6145 (2016).

**Appendix**

**Original gel of Figure S1 (editorial request)**

Based on the spectrophotometer estimates, U87MG cells (all different clones) and GBP08-P0 were found to have 0.6 relative intensity of MGMT normalized for the GADPH band, as opposed to 0.8 and 1.0 of the other samples. GBP03-P1 is one of the other patient-derived cell lines that have been tested that has a very high expression of MGMT, unlike the GBP08-P0 cells which were used for this study. The Oct related samples are not relevant. These results were consistent for all the gels that have been analyzed.

**
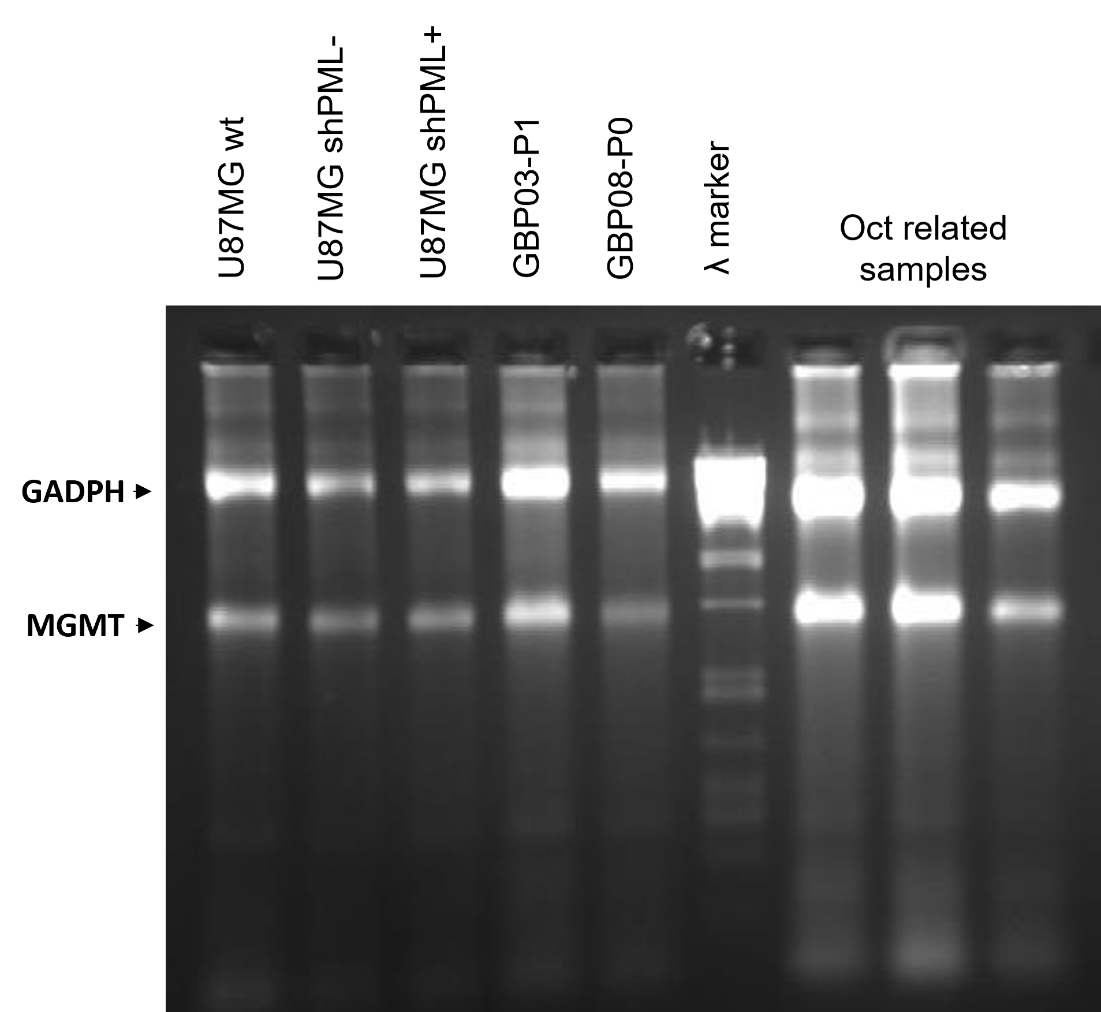
**
